# Genetic Determinants of Antagonistic Interactions and the Response of New Endophytic Strain *Serratia quinivorans* KP32 to Fungal Phytopathogens

**DOI:** 10.3390/ijms232415561

**Published:** 2022-12-08

**Authors:** Daria Chlebek, Valeriia Grebtsova, Artur Piński, Joanna Żur-Pińska, Katarzyna Hupert-Kocurek

**Affiliations:** Institute of Biology, Biotechnology and Environmental Protection, Faculty of Natural Sciences, University of Silesia in Katowice, Jagiellońska 28, 40-032 Katowice, Poland

**Keywords:** biocontrol, endophytes, fungal phytopathogens, genome analysis, gene expression, *Serratia quinivorans*

## Abstract

Fungal phytopathogens are challenging to control due to their penetration into plant tissues. Therefore, plant-colonizing bacteria could serve as an excellent weapon in fighting fungal infections. In this study, we aim to determine the biocontrol potential of the new endophytic strain *Serratia quinivorans* KP32, isolated from the roots of *Petroselinum crispum* L.; identify the related mechanisms; and understand the basis of its antagonistic interaction with taxonomically diverse fungi at the molecular level. The KP32 strain presented biological activity against *Rhizoctonia solani*, *Colletotrichum dematium*, *Fusarium avenaceum*, and *Sclerotinia sclerotiorum*, and its ability to inhibit the growth of the phytopathogens was found to be mediated by a broad spectrum of biocontrol features, such as the production of a number of lytic enzymes (amylases, chitinases, and proteases), siderophores, volatile organic and inorganic compounds, salicylic acid, and N-acyl-homoserine lactones. The higher expression of chitinase (*chiA*) and genes involved in the biosynthesis of hydrogen cyanide (*hcnC*), enterobactin (*entB*), and acetoin (*budA*) in bacteria exposed to fungal filtrates confirmed that these factors could act in combination, leading to a synergistic inhibitory effect of the strain against phytopathogens. We also confirm the active movement, self-aggregation, exopolysaccharide production, and biofilm formation abilities of the KP32 strain, which are essential for effective plant colonization. Its biological activity and colonization potential indicate that KP32 holds tremendous potential for use as an active biopesticide and plant growth promoter.

## 1. Introduction

Bacteria from the genus *Serratia* are Gram-negative rods from the *Enterobacteriaceae* family isolated from different environments, such as soil, water, plants, insects, and vertebrates [1]. It is a highly diverse group that includes biologically and ecologically various species, from those beneficial to economically important plants to pathogenic species harmful to humans [2,3]. A significant group constitutes the entomopathogenic strains of *Serratia*, known for their ability to infect insects, including various pests [4,5]. The members of *Serratia* can produce a range of biologically active compounds as part of their adaptation to ecological niche(s) [1,6], and can enhance crop yields and environmental balance in agroecosystems by facilitating the uptake of nutrients from the environment [3]. These features make *Serratia* an attractive candidate for biocontrol [6], providing cost-effective and environmentally friendly pest and pathogen control in many crops [7]. Fungal phytopathogens are challenging to control, due to their diverse host spectra and penetration into the internal tissues of plants [8,9]. Therefore, protection against fungal infection mediated by endophytic biological control agents (BCAs) establishing a stable relationship with the plant, provides additional advantages over rhizospheric or epiphytic microorganisms [10]. An improved understanding of bacterial–fungal interactions in the rhizosphere and endosphere should assist in successfully applying bacteria as BCAs against fungal pathogens of plants, providing alternatives to chemicals in sustainable agriculture.

Data in the literature have indicated that the biocontrol of plant pathogens is a complex process, and antagonism against the target fungus is often multi-faceted [11]. The mechanisms of fungal suppression include antibiosis [6], the production of lytic enzymes (e.g., chitinases, cellulase, β-1,3-glucanases, protease, DNase), siderophores [11], and volatile organic/inorganic compounds [12]. Additionally, bacteria-colonizing plant tissues may lead to induced systemic resistance (ISR), enhancing the plant’s defensive capacity against diverse phytopathogens [13]. Among the bacterial factors capable of ISR induction, the most relevant are cell surface elements (e.g., lipopolysaccharides, exopolysaccharides, flagella), acetoin, 2,3-butanediol, lytic enzymes, and antibiotics [12]. It is worth noting that numerous strains of the genus *Serratia* are considered as reservoirs of structurally unique and biologically significant novel secondary metabolites with potent antifungal activities [3], such as prodigiosin, serrawettins [14], pyrrolnitrin, and several N-acyl-homoserine lactones (N-AHLs) [15] and, therefore, have been touted as an essential tool for the effective inhibition of growth of several pathogens [10], including *Rhizoctonia solani*, *Fusarium culmorum*, *Sclerotinia sclerotiorum*, *Botrytis cinerea*, *Colletotrichum camelliae*, *Pythium ultimum*, and *Verticillium dahlia* [16]. For example, *S. plymuthica* strain IC14 protected cucumber seedlings against the *B. cinerea* grey mold and *S. sclerotiorum* white mold diseases of leaves under greenhouse conditions [17]. In the study described by Neupane et al. [18], an endophytic strain *S. proteamaculans* S4, isolated from rapeseed roots, was used as a biocontrol agent against *V. dahlia* and *R. solani*. In another study, *S. marcescens*, by inhibiting the growth of mycelium and conidia, showed the ability to biocontrol *Colletotrichum gloeosporiodes*, a causative agent of fruit anthracnose in plants [19]. Wang et al. [10] have reported that the antagonistic activity of *S. proteamaculans* 336x against *Gaeumannomyces graminis* var. tritici resulted from chitinase production. Purkayasth et al. [11] have reported that the in vitro biological activity of *S. marcescens* ETR17 against nine fungal pathogens (including *C. camelliae* and *R. solani*) was associated with the production of antibiotics (pyrrolnitrin and prodigiosin), chitinases, cellulases, proteases, and lipases, as well as siderophores. Based on the literature, the studies conducted to investigate bacterial biocontrol activities have mainly focused on the biochemical characterization of bacterial strains and evaluation of their biocontrol potential through the in vitro antagonism tests. The conventional polymerase chain reaction (PCR) method used to identify functional genes is commonly used to detect and assess the potential of corresponding antimicrobial compound syntheses. However, little is known about changes in the expression of bacterial genes following their interaction with phytopathogens, which can provide valuable clues for elucidating their biological control mechanisms. A thorough understanding of the antimicrobial mechanisms is essential for efficient and long-lasting biocontrol.

Many *Serratia* species—especially *S. protemaculans*, *S. plymuthica*, S. *marcescens*, *S. ficaria*, *S. liquefaciens*, *S. grimesii*, *S. nematodiphila*, *S. rubidaea*, and *S. fonticola*—have been well-recognized for their ability for biological control and to stimulate plant growth [2,13,17,20,21]. In contrast, endophytic bacteria belonging to the *S. quinivorans* species are still poorly characterized, and their biocontrol potential requires further understanding. In this work, a new endophytic *S. quinivorans* KP32 strain, isolated from roots of *Petroselinum crispum* L., is investigated, in order to identify the mechanisms determining the high biocontrol activity of this strain, as well as to understand the basis of the antagonistic interaction between the KP32 strain and taxonomically diverse fungal pathogens at the molecular level. Genome analysis of this strain provided opportunities to expand our knowledge of genes and their role in determining antagonistic behavior. At the same time, the quantitative reverse transcription PCR (RT-qPCR) method was used to determine the expression of functional genes. To the best of our knowledge, this is the first study of the genome of endophytic strain *S. quinivorans*, which can play a crucial role in the biological control of common fungal phytopathogens. The main objectives of the study were the following: (1) determination of the antifungal activity of the KP32 strain against phytopathogens; (2) identification of genes essential for biocontrol activity in the KP32 strain genome; (3) determination of the effect of fungal phytopathogens on the expression of genes responsible for antifungal activity; and (4) verification of the biocontrol features of the KP32 strain through a biochemical assay. We hypothesized that: (1) the KP32 strain is able to inhibit the growth of diverse phytopathogenic fungi; (2) the strain displays various mechanisms of biological activity towards fungal pathogens; and (3) the pathogens differently influence the expression of genes determining the biological activity of the tested strain.

## 2. Results

### 2.1. In Vitro Inhibition of Phytopathogens by the S. quinivornas KP32 Strain

**Figure 1:**
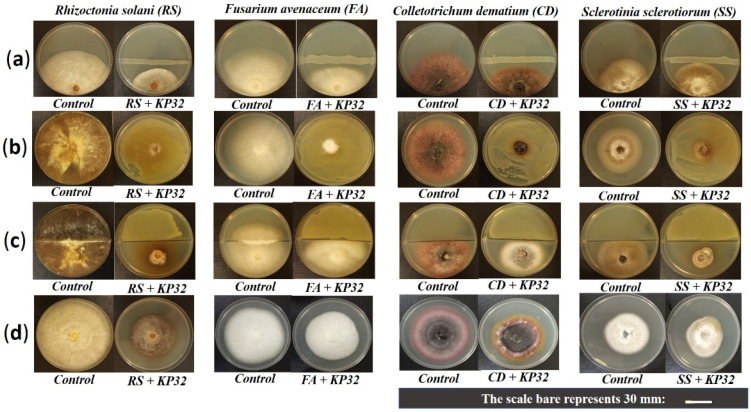
The inhibitory effect of *S. quinivorans* KP32 against fungal phytopathogens: Antagonistic action of the KP32 strain against fungal pathogens in the dual-culture assay (**a**); inhibition of fungal growth by diffusible metabolites produced by KP32 (**b**); the effect of VOCs produced by KP32 on the mycelial growth of phytopathogens (**c**); and the effect of cell-free filtrate of KP32 culture on the mycelial growth of fungal pathogens (**d**).

**Figure 2:**
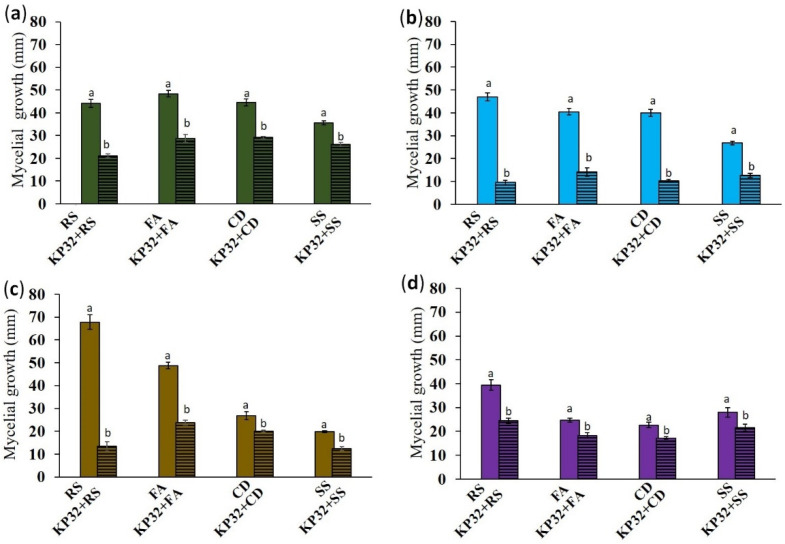
Inhibition of mycelial growth by the *S. quinivorans* KP32 strain: Mycelial growth inhibition by the KP32 strain in dual-culture assay (**a**); mycelial growth inhibition by diffusible metabolites produced by KP32 (**b**); mycelial growth inhibition by the production of VOCs by KP32 (**c**); and mycelial growth inhibition by cell-free filtrate obtained from the KP32 strain culture (**d**). All experiments were performed in triplicate. To compare the effect of the tested strain against phytopathogenic fungi, Student’s *t*-test was used. Different letters indicate a significant difference (*p* < 0.05) between the treatment and control.

*S. quinivorans* KP32 was isolated from surface-sterilized roots of *Petroselinum crispum* L. Based on the ability of the strain to degrade organic pollutants (unpublished data) and the ease of cultivation under laboratory conditions, we tested its biocontrol ability with respect to common fungal phytopathogens from four different species (Figure 1a). The KP32 strain showed potent activity against *R. solani* W70 (52.46 ± 3.62%) and moderate activity against *C. dematium* K (34.62 ± 2.08%) and *F. avenaceum* (40.43 ± 3.75%). It exhibited the lowest ability to inhibit the growth of *S. sclerotiorum* (26.41 ± 7.91%); see Figure 2a. The diffusible compounds produced by the KP32 strain exhibited significant antifungal activity against all tested phytopathogens (Figure 1b). Mycelial growth of *R. solani* W70 was inhibited up to 79.36 ± 2.72%, relative to the control (Figure 2b). Besides active diffusible compounds, the KP32 strain also produced volatile organic compounds (VOCs), inhibiting the mycelial growth of fungal phytopathogens (Figure 1c). In this study, the KP32 strain showed maximum antifungal activity with 80.37 ± 2.26% of mycelial growth inhibition in *R. solani* W70 compared to control, due to the production of active VOCs (Figure 2c). The production of both the diffusible and volatile compounds may be a critical phenomenon presented by the KP32 strain, which plays a crucial role in the in vitro inhibition of tested pathogens. The antifungal activity of supernatant obtained from the KP32 strain culture was also verified. The results of this experiment provide evidence that extracellular metabolites in the supernatant of the KP32 strain inhibit the growth of pathogens. Moreover, in each of the tests, apart from growth inhibition, changes in the mycelial morphology of all fungal pathogens were observed (Figure 1d and Figure 2d).

### 2.2. The Properties of Genome and Phylogenetic Analyses

A whole-genome sequence analysis was performed following the determination of the antagonistic activity of the KP32 strain. The newly sequenced 5,456,872 bp genome of *S. quinivorans* KP32 (Table 1) was assembled into 107 contigs with an average G + C content of 64%. There were about 5098 predicted protein-encoding sequences (CDSs). In addition, the KP32 strain genome included 79 tRNA and 35 rRNA genes. The functional analysis performed using the Kyoto Encyclopedia of Genes and Genomes (KEGG) identified 3342 genes (65.5% of all CDSs) involved in any of the metabolic pathways included in the knowledge base (Figure S1a). From those genes that were classified among the KEGG pathway categories, the most significant number was involved in the metabolism of carbohydrates (10.32%), amino acids (5.98%), and cofactors and vitamins (4.37%). It is worth emphasizing that the presence in the genome of the biosynthetic pathway for the production of aromatic amino acids majorly contributes to the broad functional spectrum of organisms in nature, as various pigments, siderophores, antibiotics signaling compounds, structural compounds, defense metabolites, and other secondary metabolites are derived from this pathway [22]. Most of the remaining genes were involved in environmental and information-processing processes, such as membrane transport (7.78%) and signal transduction (4.70%). It is worth noting that a number of genes were involved in the biosynthesis of secondary metabolites and metabolism of terpenoids and polyketides, which represent essential bioactive natural products with a broad range of biological activities (Figure S1b). These compounds have general inhibitory activity against various pathogens, including fungi [6]. A total of 5018 protein-encoding genes were assigned to 24 clusters of orthologous genes (COGs); see Figure S2. These results confirmed a preference for the metabolism and transport of carbohydrates (8.37%) and amino acids (9.63%). Other high-percentage clusters represented genes involved in energy production and conversion (5.66%), as well as cell wall/membrane/envelope biogenesis (5.88%).

The production of metabolites was predicted with antiSMASH. In the KP32 strain, seven gene clusters were identified (Table S1). Of these, two were non-ribosomal peptide-synthetase (NRPS)-type gene clusters, predicted for the biosynthesis of amonabactin, enterobactin, and streptobactin; two clusters were designated as the NRPS-like predicted for the biosynthesis indiogine; and the other three were predicted to be engaged in the synthesis of betalactones, nucleosides, and siderophores (e.g., arylpolyene and aerobactin). These clusters presented similarity to the known clusters determining the production of secondary metabolites, which play an important role in the suppression of pathogens. The structure of these secondary metabolites was predicted using the PubChem website (https://pubchem.ncbi.nlm.nih.gov/, accessed on 9 November 2021), and is furnished in Supplementary Figure S3.

**Figure 3:**
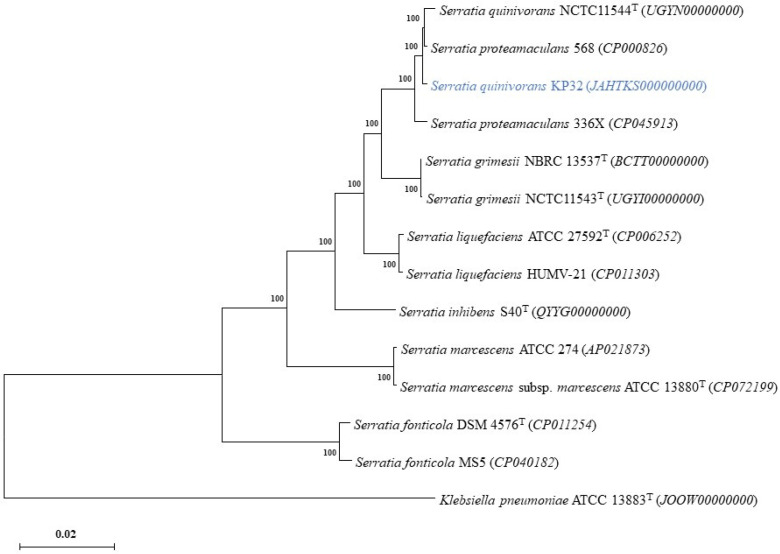
Phylogenetic tree of *S. quinivorans* KP32 (marked in blue) based on the alignment of the core proteome of 14 strains, with *Klebsiella pneumoniae* ATCC 13883^T^ as an outgroup. The accession numbers of genomes are indicated in brackets. Branching percentage values were calculated with the use of the 1000 bootstraps resampling test. The tree was constructed using the maximum likelihood method, and the scale bar represents 1% nucleotide sequence divergence.

The phylogenetic tree of *Serratia quinivorans* KP32, based on the alignment of the core proteome of 14 strains with *Klebsiella pneumoniae* ATCC 13883T as an outgroup, is illustrated in Figure 3. In the phylogenetic group of the genus *Serratia*, KP32 was grouped closely with *S. quinivorans* NCTC11544, *S. proteamaculans* 568, and *S. proteamaculans* 336X.

### 2.3. Genes Essential for Biocontrol Activity in the KP32 Strain Genome

Genome analysis (Table S2) revealed the presence of genes involved in the biosynthesis and transport of siderophores, such as catecholates (enterobactin) and hydroxymates (aerobactin, ferrichrome), including *entC*, *entB*, and *entA* genes encoding enzymes that catalyze the formation of dihydroxybenzoic acid (DHBA) from chorismite. We also found genes encoding multi-enzyme complexes composed of the products of *entD*, *entE*, *entF*, and *entG*, which catalyze the synthesis of enterobactin from three molecules each of DHBA and serine. As for genes involved in aerobactin synthesis, the *iucABCD* gene cluster was identified, and the gene coding for the receptor of this siderophore (*iutA*) was also found upstream. Besides genes related to siderophore biosynthesis, genes encoding the ABC transporter complex associated with aerobactin production were also identified. Additionally, the KP32 strain was found to carry genes encoding ferrous iron uptake systems (*efeUOB*, *feoABC*, *fepABCDG*, and *fhuBCD*) and a number of ABC transporters and receptor proteins of major facilitator superfamily (MFS) of transporters. We also found two copies of the *dps* gene related to iron storage inside the cell and proteins that present Fe-S-based prosthetic groups as those coded by *sufEDBCA*. This suggests that the KP32 strain can uptake iron, importing and exporting it to the host plant. The KP32 strain also carried phosphate transporters, such as the *pstABSC* transporter system. Furthermore, we found genes involved in phosphonoacetate degradation (*phnA*), polyP formation (*ppk*), genes encoding phosphatases (*ppa*), and two-component systems (*phoB*, *phoR*, *phoA*, *phoU*, *phoH*) responsible for the phosphate starvation response. Genome analysis revealed the presence of *gcd* for gluconic acid. We also searched for indole-3-acid (IAA) biosynthesis pathways in the KP32 genome. The analysis revealed the presence of biosynthesis genes in the IPyA pathway (*ipdC*, *aspC*, *aldA*) and tryptophan (TRP) biosynthesis genes. It is worth emphasizing that the KP32 strain also carried operons *speAB* and *speDE*, which are involved in spermidine biosynthesis. Genes required for assimilatory sulfate reduction (H_2_S production) were present in the KP32 genome (*cysND*, *cysC*, *cysH).* Additionally, genes encoding the enzymes cystathionine β synthase (CBS) and cystathionine-γ-lyase (CTH), which are involved in other possible pathways for H_2_S production, were also present. Additionally, the KP32 strain possessed the genes involved in acetoin (*ilvM*, *ilvH*, *ilvB*, *budA*) and 2,3-butanediol (*butA*) production. In addition, the genes for synthesis of VOCs, such as 4-hydroxybenzoate (*ubiC*), methanethiol (*met*), and isoprene (*gcpE* and *ispE*), were identified. The genome of the KP32 also carried the gene *hcnC* and genes for regulatory cascades such as GacS/GacA, as well as auto-regulators, which are involved in the biosynthesis and transportation of antibiotics [23]. In the genome of the KP32, we also found a gene encoding mannitol dehydrogenase (*mtlK*).

In the genome of the KP32 strain, we confirmed the presence of a gene encoding lytic and antioxidant enzymes. We found two genes encoding chitinases and enzymes involved in further chitin degradation. Furthermore, the analysis of the genome revealed genes involved in the production of amylase (*amyA*), lipase (*apeE*), and proteases (*yhbV*, *yegQ*). Additionally, we found a wide range of enzymes and regulators that help bacteria to cope with oxidative stress, such as *katA* (catalase, CAT), *katG* (catalase-peroxidase), *sodA*, *sodB*, *sodC* (superoxide dismutase, SOD), *gstA* (glutathione S-transferases, GTS), *gpo* (glutathione peroxidases), *gsiABCD* (glutathione ABC transporter), and *gor* (glutathione reductase). Additionally, genes coding for the hydrogen peroxidase sensor OxyR and the stress response sigma factor RpoS were identified. Furthermore, we also identified several genes (*uspABCEFG*) encoding universal stress proteins.

Bacterial colonization is dependent on motility and chemotaxis for attractive tags that may be compounds that activate bacterial-specific signaling pathways. In the genome of the KP32 strain, a cluster of genes associated with the induction of hyper-adherence (*yidE-16hspA-yidR-yidQ*), which has been described as fundamental for some microorganisms to colonize plants and seeds, was identified [24]. Moreover, we confirmed the presence of many genes involved in the biosynthesis and assembly of flagella, such as the *flg*, *fli*, and *flh* operons, as well as genes coding for the flagellar motor proteins MotA and MotB. Additionally, many genes involved in chemotaxis (*cheBWARZ*, *tsr*, *aer*, *tar*, and *mcp*) and adhesive structure, which play significant roles in plant host–bacteria interactions, were recognized. We also found genes involved in the biosynthesis and assembly of the type IV pilus system (T4PS) (*hofBCMOPQ*) and pilin (*ppdABC*). The analysis of the KP32 genome also revealed several genes involved in cellulose (*bcsABZC* and *bcsEFG* operons) and colonic acid biosynthesis (*wza wzb*, *wzc*). These exopolysaccharides are produced by many *Enterobacteriaceae* and are critical for biofilm formation [25]. The genome of KP32 carried a *csgG* gene involved in curli fiber biosynthesis, which plays an essential role in adhesion to surfaces, cell aggregation, and biofilm formation [26]. Bacterial biofilms can be regulated by a mechanism that uses small signaling molecules (called autoinducers) for cellular communication, called *quorum sensing* (QS). The KP32 strain carried genes (*tqsA*, *luxS*) encoding enzymes that catalyze the synthesis of the signal precursor for autoinducer-2 mediated QS [27]. In the KP32 genome, we also identified 162 putative genes encoding carbohydrate-active enzymes (CAZy) distributed unevenly among the five CAZy families (Table S3). CAZymes with the potential to degrade many cell wall polymers, including hemicellulose, pectin, polysaccharides, and peptidoglycans, were found (Table S4).

Protein secretion plays a central role in modulating the interactions of bacteria with their environments. Different protein secretion systems are activated in some bacteria species during interactions with roots and pathogens [28]. Through these systems, bacteria secrete antibiotics, secondary metabolites, enzymes, toxins, and peptides into the surrounding environment, compete with nearby microorganisms, or are incorporated and used by their host plant [29,30]. In the genome of the KP32, we identified 30 genes involved in bacterial secretion systems. Among them, the Sec and Tat secretion pathways and most of the type II secretion systems (T2SS) and type VI secretion systems (T6SS) were found. It is worth emphasizing that more than one type of secretion system in the genome is evidence that the KP32 strain possesses efficient translocation systems for secreted compounds across the inner and outer membranes of cells. T6SSs and T2SSs are fairly well-conserved in a wide range of Gram-negative bacterial species. T2SSs transport folded proteins from the periplasm into the extracellular environment. This system secreted proteins, which have a range of biological functions: generally enzymes, such as lipases, proteases, phosphatases, and several proteins that process carbohydrates [30]. Interestingly, in 2018, Trunk et al. [31] reported the type VI secretion system-mediated delivery of antifungal effector proteins between *Serratia marcescens* and fungal cells.

The genome of the KP32 strain also carried horizontally transferred genomic islands (GIs). A GI often carries genes essential for genome evolution and adaptation to the environment. The GIs comprised 440 genes, mainly encoding various proteins primarily involved in multi-drug resistance regulatory factors (e.g., RhaR, SutR, RfaH, HxIR, ExaE) and heat-shock tolerance. We also found phage protein genes in the GI, indicating previous infection by phages. Moreover, no pathogenicity factor or compound has been linked to the KP32 genome, and the absence of any virulence, resistance, and pathogen-associated genes was confirmed.

### 2.4. Determination of the Effect of Fungal Phytopathogens on the Expression of Genes Responsible for Antifungal Activity

**Figure 4:**
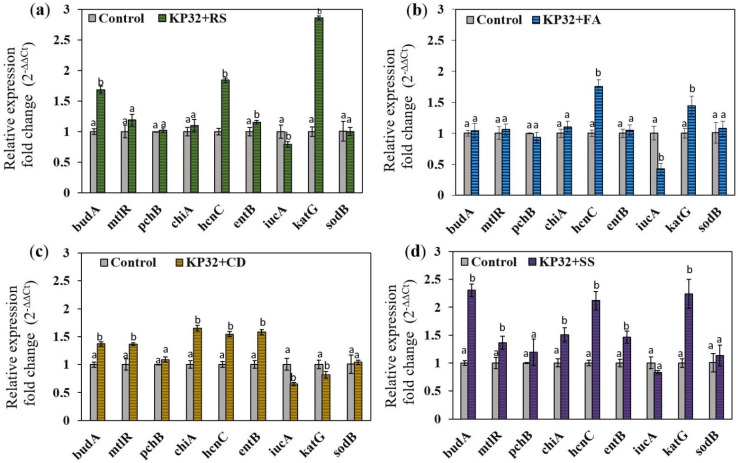
Analysis of the expression of genes potentially engaged in the biocontrol activity of the KP32 strain in the presence of fungal pathogens analyzed with fungal filtrates after 96 h: (**a**) *Rhizoctonia solani* (RS); (**b**) *Fusarium avenaceum* (FA); (**c**) *Sclerotinia sclerotiorum* (SS); and (**d**) *Colletotrichum dematium* (CD). Fold change in gene expression was evaluated by RT-qPCR, according to the 2^−∆∆CT^ method; same letters indicate no statistically relevant difference between the treatments (*n* = 6), as determined by Student’s *t*-test (*p* < 0.05).

While genome sequencing and analyses revealed the presence of genes engaged in biocontrol mechanisms, studies on their expression are essential to understand the relationship between the interacting bacteria and specific fungal pathogens. For this study, several genes encoding features determining antifungal activity [11,12] were selected and analyzed using RT-qPCR. Total RNA was isolated from the KP32 cultures treated individually for 96 h with filtrates of *R. solani*, *F. avenaceum*, *C. dematium*, *S. slecrotiorum* and untreated bacteria (control). The changes in bacterial gene expression in response to the filtrates are summarized in Figure 4. We observed that studied phytopathogens differently influenced the expression of genes determining the biological activity of KP32. The gene expression profiles revealed mainly up-regulation of genes related to the biosynthesis of acetoin (*budA*), hydrogen cyanide synthase (*hcnC*), chitinase (*chiA*), mannitol dehydrogenase (*mtlR*), bifunctional isochorismate lyase (*entB*), and catalase (*katG*). The expression levels of the *budA*, *hcnC*, and *katG* genes were significantly up-regulated after the exposure of the KP32 strain to the fungal filtrate of *R. solani* W70, where the expression was 1.7-, 1.85-, and 2.87-fold higher than in the control, respectively. The up-regulation of *hcnC* gene was also confirmed for other treatments. It is worth noting that the highest expression for the *hcnC* gene was observed for the KP32 culture inoculated with the fungal filtrate of *S. sclerotiorum*, where the expression was 2.12-fold higher than in the control. The expression level of the *chiA* gene increased in the KP32 cells treated with fungal filtrates of *C. demaitum* and *S. sclerotiorum*, being 1.5- and 1.65-fold higher than in the control, respectively. In the presence of the filtrates of these fungi, increased expression of the *entB* gene was also observed. It is worth emphasizing that the highest expression of the *budA* gene was detected for the KP32 culture inoculated with fungal filtrate of *S. sclerotiorum*, with expression 2.3-fold higher than in the control. The *iucA* gene encoding aerobactin synthase was down-regulated when fungal filtrates were added to the culture of the KP32 strain. In addition, the presence of fungi in the cultures did not change the expression of *pchB* and *sodB* genes, while the expression of *katG* was significantly upregulated after culturing the KP32 in the presence of *R. solani*, *F. avenaceum,* and *S. sclerotiorum* filtrates. The obtained results showed that the production of acetoin and hydrogen cyanide contribute to the biological activity of the KP32 strain towards each tested phytopathogen and the reduction of oxidative stress caused by the fungal pathogens could be related to the increased catalase production by the strain.

### 2.5. Evaluation of Biocontrol Features of the KP32 Strain in a Biochemical Assay

Once the effective antifungal activity by the tested strain was demonstrated and the genetic analysis revealed crucial genes for biocontrol activity, the mechanisms potentially involved in biocontrol and plant growth promotion were evaluated using the biochemical test. The KP32 strain formed an orange halo zone on medium chrome azurol S (CAS) agar and a clear zone formation on Pikovskaya’s agar media, indicating siderophore production and phosphate solubilization. The KP32 strain produced VOCs such as acetoin and 2,3-butanediol. It was also able to produce IAA at the concentration of 14.32 ± 0.12 μg/mL in 1% tryptophane medium and salicylic acid (SA) at the concentration of 5.43 ± 0.89 μg/mL in succinate medium. After 36 h of incubation at 30 °C, the KP32 strain metabolized 3 mM 1-aminocyclopropane-1-carboxylate (ACC) as the sole source of nitrogen in Dworkin and Foster (DF) medium. It also presented ammonia and hydrogen cyanide (HCN) production. The most important mechanisms to explain the beneficial effects of KP32 included the production of lytic enzymes, which have an excellent biocontrol potential. These enzymes may confer protection against a wide range of phytopathogens. In this study, we observed that the KP32 strain produced proteases, amylases, and chitinases in the absence and presence of fungal cell-free supernatant (Table 2). Exposure of the KP32 strain to the filtrate of *R. solani* W70 resulted in a noticeable increase in protease activity, from 10.32 ± 0.45 to 24.03 ± 0.21 U/mL. Meanwhile, the addition of *S. sclerotiorum* filtrate resulted in decreased protease activity (4.98 ± 0.24 U/mL), compared to the control. The maximum activity of chitinase (2.12 ± 0.1 U/mL) was recorded in the presence of cell-free supernatant of *C. dematium* K. The activity of chitinase in the absence of fungal supernatant was lower (0.84 ± 0.12 U/mL). The KP32 strain did not produce cellulase in the absence as well as the presence of fungal filtrates. The statistical analyses indicated that fungal cell-free supernatants did not influence the increase in amylase activity. To study the impact of phytopathogens on the generation of oxidative stress in the KP32 strain, the activity of enzymatic antioxidants, including superoxide dismutase and catalase, were measured. The KP32 strain exhibited a higher CAT activity after exposure to cell-free supernatant. Interestingly, CAT was the most sensitive to *R. solani* W70 supernatant. The calculated SOD activities led to the conclusion that SOD in the KP32 strain was less active than CAT after exposure to fungal cell-free supernatant.

The colonization of plants by endophytic bacteria seems to be essential for disease management and plant growth improvement. The KP32 strain showed various features crucial for plant host colonization (Table 3). Motility assays revealed its ability to spread on 0.3% (swimming), 0.5% (swarming), and 1% (twitching) agar plates. The tested strain produced exopolysaccharide (EPS), as evidenced by smooth black colonies with a slimy and shiny surface on Congo Red Agar (CRA) medium. It was also demonstrated that the KP32 strain can produce N-AHLs, as confirmed by a dark brown color in the sample. The obtained results exhibited a weak ability of the KP32 strain to self-aggregate, based on the classification described in [32]. After 24 h of incubation, the autoaggregation of KP32 reached 34.2 ± 0.09%. Another important feature is the ability of the strain to form a biofilm. The results indicated that KP32 is a moderate biofilm producer [33]. In this study, we demonstrated that the KP32 strain can grow in the presence of a wide spectrum of organic compounds as the sole source of carbon and energy (Table S5). Organic compounds, including organic acids (e.g., malic, citric, fumaric, and succinic) produced by plants, act as chemotactic agents to recruit beneficial bacteria to the rhizosphere. The chemotactic response of bacteria to organic acids is crucial for the effective colonization of plant tissues [34].

## 3. Discussion

Microorganisms can limit the adverse effects of pathogens. This inhibition of pathogenesis is known as biocontrol and has shown promise as a practical alternative for controlling plant pathogens within sustainable agriculture systems [35]. Many biocontrol agents are selective to host species, type of pathogen, environmental conditions, soil types, and seasons [35]. Therefore, systematic analyses are required to discover new biocontrol agents that can respond to a wide range of environments [36]. To benefit their host, bacteria must possess a number of direct and/or indirect mechanisms and activities, allowing them to competitively colonize the plant [37]. To date, some strains of the genus *Serratia* have been shown to control an extensive range of phytopathogens and facilitate growth enhancement in several crops [25].

Despite the existence of knowledge regarding the ecology and pathogenicity mechanisms of *S. marcescens*, *S. plymuthica*, and *S. proteamaculans*, *S. marcescens* N1-14, SR-1, and TRS-1; *S. plymuthica* A21-4 and IC14; and *S. proteamaculans* 1-102 and 3Rc15 are the most-studied as biological plant protection agents [16,38,39,40,41,42,43,44,45]. To the best of our knowledge, little is known about the biocontrol activity of *Serratia* strains originally isolated from the internal tissues of plants. For this study, we characterized an endophytic bacterium—the *S. quinivorans* KP32 strain—which exhibits biocontrol activities against a variety of phytopathogens. It is worth noting that genome analysis and RT-qPCR experiments opened up several prospects to understand the mechanisms used by this endophytic bacterium for the biocontrol of pathogenic fungi.

We found that the KP32 strain, which is closely related to *S. quinivorans* NCTC11544 and *S. proteamaculans* 336X—biological control agents of wheat take-all [10]—can counter the growth of selected fungal pathogens. The studied strain inhibited the growth of *R. solani* W70 to the greatest extent, which is even more important, considering that *R. solani* represents an essential group of soil-borne basidiomycete pathogens, which cause typical symptoms of pre-emergence and post-emergence damping-off, root rot, or stem canker [46,47]. Our results demonstrated that the KP32 strain may be used as an effective BCA against this pathogen. Apart from the inhibitory effect of the KP32 on the growth of the selected fungi, macroscopic changes in the *R. solani* W70 mycelium were noted, which confirmed the biological activity of the KP32 strain. Similar results have been observed by Purkayastha et al. [11]. In the interaction zone of *S. marcescens* ETR17 with *R. solani*, deformation, bulging, and lysis of the mycelium was confirmed through scanning electron microscopy (SEM). Gkarmiri [48] has revealed the effects of the strains *S. proteamaculans* S4 and *S. plymuthica* AS3 on the morphology of *R. solani* mycelium, as well as details of the inhibition of fungal growth potential through the production of chitinases, proteases, and antifungal compounds. The studied KP32 strain also inhibited the growth of *C. dematium* K, *F. avenaceum*, and *S. sclerotiorum* K2291. Mycelial growth and conidial germination caused by *Colletotrichum gloeosporioides* were inhibited by *S. marcescens* CFFSUR-B2, CFFSUR-B3, and CFFSUR-B4 [19]. On the other hand, Li et al. [49] have indicated the ability of *S. marcescens* FS14 to inhibit the growth of *Sclerotinia sclerotiorum* and *Fusarium oxysporum* without direct contact. In addition, according to the authors, the changes in the mycelia of pathogens observed in dual culture tests may have resulted from the ability of the strain to secrete extracellular metabolites [49].

Many publications have highlighted the phenomenon of the effects of volatile bacterial compounds on plants and fungi [50]. It is known that *Serratia* species produce different VOCs that play a role in the plant–microbe interactions [51]. In this study, we also revealed that the production of VOCs by the KP32 strain was the most crucial mechanism in the in vitro inhibition of the tested phytopathogens—mainly *R. solani* W70 (even up to 79.36 ± 2.72%). In other research, Marzouk et al. [47] have provided evidence regarding the VOC-mediated biocontrol ability of seed-borne endophytic bacteria to reduce *R. solani* seedling damping-off and fruit rot of tomato, making them valuable agents for pre- and post-harvest control of this disease. It is worth noting that most volatiles are products of bacterial metabolism, such as fermentation, amino acid degradation, sulfur metabolism, and fatty acid biosynthesis [52], which were also abundant pathways in the KP32 genome. We found that the KP32 strain could produce acetoin and butanediol, two well-known volatile compounds that act as growth-promoting factors and which increase plant resistance against pathogens [53]. It is worth emphasizing that the biosynthesis pathway of these compounds was recognized in the genome of the KP32 strain. Furthermore, in this study, we observed up-regulation of the *budA* gene involved in the biosynthesis of acetoin in the KP32 culture inoculated with fungal filtrates, suggesting that this mechanism is related to the antifungal properties of the bacterium. Numerous studies have shown that the production of acetoin and 2,3-butanediol by *S. proteamaculans* 568 stimulated growth and systemic resistance in plants by inducing expression genes in the signaling pathways of SA, jasmonic acid, and ethylene [54,55,56]. In turn, high acetoin levels in the mutant strain of *Bacillus velezensis* resulted in a significant increase in the production of H_2_O_2_, an increase in the activity of enzymes related to the defence response, and callose deposition in *A. thaliana* [57]. Furthermore, several sulfur modulation pathways were also present in the KP32 genome, which may account for its ability to produce sulfur-based volatiles, like dimethylsulfide and H_2_S, that can improve plant growth and root colonization [52], as well as inhibiting fungi spore germination, germ tube elongation, and mycelial growth [58]. The genome of the KP32 strain also carried several genes involved in fatty acid biosynthesis and modification, which play roles in the production of volatile ketones and alcohols. Among the volatile substances inhibiting microorganism growth, the inorganic volatile compound HCN might also have toxic effects on various fungal and bacterial plant pathogens [59]. Moreover, HCN can indirectly increase phosphorus and iron availability to plants, resulting in increased plant growth promotion activity [60]. This compound inhibits electron transport for energy supply to cells, leading to the organism’s death. It also affects the proper functioning of enzymes and natural receptors through reversible inhibition mechanisms [61]. The gene required for the production of HCN was identified in KP32 genome. It is worth noting that the expression level of the *hcnC* gene in the KP32 strain was significantly up-regulated after exposure to fungal filtrates of all tested pathogens, suggesting that the production of hydrogen cyanide is the primary mechanism in their inhibition. In addition, we confirmed the ability of KP32 to produce HCN in biochemical tests.

Another mechanism involved in the antifungal activity of the KP32 strain is the production of lytic enzymes. This function is essential for the antagonism of biocontrol agents against various phytopathogens. The data in the Literature have indicated the suppressive effects of *Serratia* strains on pathogen growth and/or fungal disease to be generally correlated with high chitinase activity [62]. Bacterial chitinases can compromise fungal spore integrity and generate germ tube abnormalities [25]. Furthermore, chitinase promotes the degradation of mycelia of several pathogenic fungi, including *Rhizoctonia*, *Fusarium*, *Botrytis*, and *Sclerotium* [63,64]. Apart from chitinase, extracellular proteases play a crucial role in inhibiting the growth of fungi; for example, *S. plymutica* IC14 exerted an antifungal activity against *S. sclerotiorum* and *B. cinerea* through the production of proteases [63]. It is worth noting that lytic enzymes may aid in intracellular colonization, develop endophytism [65], and serve as determinants in the induction of ISR in plants [66]. In this study, high chitinase activity was observed in the KP32 culture exposed to the cell-free supernatants of *C. dematium* K and *S. sclerotiorum* while, in the presence of the cell-free supernatant of *R. solani* W70, a high activity of protease was observed, suggesting that these enzymes play a crucial roles in the biocontrol of these pathogens. The lower activity of chitinase in the culture of the KP32 strain exposed to cell-free supernatants of *F. avenaceum* and *R. solani* may reflect weaker inhibition of mycelial fungal growth than in the case of *C. dematium K*. The significant role of lytic enzymes, such as chitinase, glucanase, and protease, in controlling *R. solani* by *S. fonticola* has been demonstrated by Faltin et al. [67]. In another study, Guitiérrez-Román et al. [19] have shown that *S. marcescens* CFFSUR-B2, CFFSUR-B3, and CFFSUR-B4 inhibited microfungal *Colletotrichum* genus by active production of chitinases and the antibiotic prodigiosin. Similarly, Someya et al. [64] have reported that the effectiveness of inhibition of *F. oxysporum* by *S. marcescens* B2 (Percent Growth Inhibition (PGI) about 70% compared to the control) is related to the production of prodigiosin and high chitinolytic activity.

One of the most crucial direct biocontrol mechanisms is the production of siderophores, which are involved in iron sequestering [68]. Many fungi colonize the rhizosphere and must cope with strong competition from bacteria. It has been well-documented that siderophore-producing antagonistic bacteria exhibit better rhizosphere competence and suppress plant disease by inhibiting plant fungal growth or metabolic activity [69,70]. The ability to inhibit the growth of fungi of the genera *Colletotrichum*, *Fusarium*, and *Rhizoctonia* has been observed in many siderophore-producing microorganisms [71,72], which may indicate that the ability of the strain KP32 to produce siderophores may facilitate the mechanism of *C. dematium* K, *R. solani* W70, and *F. avenaceum* biocontrol. Our study confirmed the presence of genetic determinants involved in the secretion of large numbers of diverse siderophores and the synthesis of siderophore receptors. We also demonstrated the ability of KP32 to produce siderophores in biochemical tests. In a similar study, Press et al. [73] reported that *S. marcescens* 90–166 inhibited anthracnose in cucumber, caused by *C. dematium*, through the production of catecholate siderophore and ISR induction [73]. Interestingly, in this study, we revealed an increased expression of the *entB* gene, encoding enterobactin (catecholate siderophore), when culturing the KP32 in the presence of the filtrates of *C. demetium*. In another study, Singh et al. [74] have shown that a *Pseudomonas putida* mutant with over-production of siderophores was more effective in inhibiting pathogenic *Fusarium oxysporum* in tomato.

The characterization of biocontrol strains most often includes evaluation of plant growth-promoting traits, the presence of which increases the overall quality of a strain [75]. In this study, we showed that the KP32 strain possessed growth-promoting attributes such as IAA, SA, ammonia production, and phosphate-solubilizing ability. The ability of bacteria to solubilize phosphate affects the uptake of nutrients, including calcium, potassium, iron, copper, manganese, and zinc, and can increase plant biomass [76]. For example, the potential use of *Serratia* sp. J260 and *S. phosphoticum* as bio-fertilizers has been reported, as they favor growth and plant productivity by supplying phosphorus in useful forms [77,78]. Compared to the data in the literature, the phosphate solubilization activity of the KP32 strain was moderate (PSI: 2.75 ± 0.11); similarly, the PSI of *Pseudomonas aeruginosa* KUPSB12 was 2.85 ± 0.2 [79], and that of *Burkholderia multivorans* WS-FJ9 was 2.27 ± 0.09 [80]. In turn, in the research of Pande et al. [81], in the endophytic bacteria *Alcaligenes aquatilis* and *Burkholderia cepacia*, the PSI index ranged from 4.88 ± 0.69 to 4.48 ± 0.30. Another essential feature of the KP32 strain is the SA production ability. Some plant growth-promoting bacteria (PGPB) strains produce SA, which stimulates systemic acquired resistance. The role of SA in ISR elicited by PGPB has been observed against the blue mold of tobacco [82]. In this study, we also demonstrated that the KP32 strain could produce N-AHLs involved in QS, which are employed by numerous bacteria to regulate gene expression in response to cell density. In many rhizobacteria, QS mechanisms induce the synthesis of antimicrobial secondary metabolites and extracellular lytic enzymes inhibitory against other fungi, bacteria, protozoa, and nematodes [83]. Furthermore, we also confirmed the presence of genetic determinants involved in N-AHL biosynthesis in the genome of the tested strain.

Colonization of plants has been recognized as an essential factor for BCA to survive in the rhizosphere battlefield against phytopathogens [84]. Based on this fact, we analyzed different colonization features of the KP32 strain. For successful root colonization, chemotaxis and biofilm formation are the two most crucial activities in bacteria. First, we demonstrated that the KP32 strain utilizes a wide range of organic compounds secreted from root exudates as carbon and energy sources. It is worth noting that root exudates (e.g., organic acids, enzymes, phenolics, sugars, carbohydrates, and proteins) can chemoattract and help bacteria to colonize the surface of plant roots [34]. The KP32 strain carried multiple genes responsible for the metabolism of the primary root exudates and several genes involved in the biosynthesis of EPS, such as cellulose and colonic acid, which are critical for biofilm formation [85,86]. A biofilm is a surface-linked efficient microorganism confined by a polymeric matrix, including self-making exopolysaccharides, extracellular DNA, and proteins related through the biotic surface [53]. Some PGPB have shown antagonistic activity in response to phytopathogens by starting biofilm-like assemblies, as previously reported in *Bacillus pumilus* HR10 [86] and *Burkholderia gladioli* 3A12 [87]. In another study, Mateolli et al. [25] have described the strategy deployed by *S. marcescens* UENF-22GI to hinder fungal growth, which likely involves massive biofilm formation on *Fusarium* hyphae, thus potentially facilitating the colonization and degradation of fungal cell walls. It is worth noting that genome analysis also revealed the presence of genes for flagellar biosynthesis, assembly, and chemotaxis. The ability of the KP32 strain to move enables not only chemotaxis towards plant root exudates, but also enhances the effect of biocontrol [88]. For example, Hover et al. [89] have shown that *S. marcescens* strains with a defect in flagella synthesis migrate significantly slower along the hyphae of *Rhizopus oryzae*, resulting in delayed fungal elimination. During colonization, endophytic bacteria must survive in a highly oxidative environment. In this study, we demonstrated that the KP32 strain can produce catalase, allowing it to overcome oxidative stress.

## 4. Materials and Methods

### 4.1. Bacterial Strains and Growth Conditions

*Serratia quinivorans* KP32 was isolated from surface-sterilized roots of *Petroselinum crispum* L. growing in the area contaminated with phenolic compounds around the Kalina Pond in Świętochłowice, Southern Poland (50.27894N, 18.92657E), according to the standard protocol described in [90]. The KP32 isolate was identified by sequencing of the 16S rRNA gene. The strain was grown in LB medium (LB Broth, BTL, Warsaw, Poland) for 24 h at 30 °C, and genomic DNA was extracted using a GeneMatrix Bacterial and Yeast Genomic Purification Kit (EURx, Gdańsk, Poland), according to the manufacturer’s protocol. Amplification of the 16S rRNA gene was performed with 8F (5′-AGAGTTTGATCCTGGCTCAG-3′) and 1492R (5′-GGTTACCTTGTTACGACTT-3′) universal primers. The PCR master (50 μL) contained 50 ng of genomic DNA, 1U of Taq DNA polymerase, 1× TaqDNA polymerase buffer, 0.2 mM of dNTP, 2 mM MgCl2, and 0.2 μM each of the forward and reverse primers. The PCR cycling conditions were 5 min at 94 °C; 30 cycles of 1 min at 94 °C, 45 s at 54 °C, and 90 s at 72 °C; and 10 min at 72 °C. The PCR-amplified 16S rRNA region was sequenced directly by GENOMED (Warsaw, Poland). The obtained sequences were compared with the public databases using NCBI BLASTN online (http://www.ncbi.nlm.nih.gov/, accessed on 2 June 2018). The taxonomic affiliation of the KP32 strain was also supported by the genomic sequencing. The bacterial strain used in this study was cultivated in Luria-Bertani broth (LB Broth, BTL, Warsaw, Poland) with shaking (130 rpm) at 30 °C or on LB Agar (BTL, Warsaw, Poland) at 30 °C. For the in vitro screening of antifungal activities, the KP32 strain was grown on potato dextrose agar (PDA) (A&A Biotechnology, Gdańsk, Poland) at 30 °C.

### 4.2. Pathogenic Fungi

*Rhizoctonia solani* W70 (RS), *Fusarium avenaceum* (FA), *Colletotrichum dematium* K (CD), and *Sclerotinia sclerotiorum* K2291 (SS) were derived from the Microbial Culture Collection of the Institute of Biology, Biotechnology and Environmental Protection (Faculty of Natural Sciences, the University of Silesia in Katowice, Katowice, Poland). Fungal phytopathogens were isolated from plant tissues exhibiting apparent symptoms of diseases, such as blights, spots, wilts, anthracnose, and/or rots. *Rhizoctonia solani* W70 was isolated from grapevine (*Vitis vinifera* L.), *Colletotrichum dematium* K and *Sclerotinia sclerotiorum* K2291 were isolated from caraway (*Carum carvi* L.), and *Fusarium avenaceum* was isolated from wheat (*Triticum* L.). All pathogens were incubated on PDA (A&A Biotechnology, Gdańsk, Poland) at 30 °C or in a liquid medium (pH 5.6) composed of 20 g/L of glucose, 10 g/L of peptone, 10 g/L of yeast extract, 0.5 g/L of Mg_2_SO_4_ × 7 H_2_O, and 1 g/L of K_2_HPO_4_ at 30 °C.

### 4.3. In Vitro Screening of Antagonistic Behavior of the KP32 Strain against Fungal Phytopathogens

#### 4.3.1. Dual-Culture Assay

The KP32 strain was screened for its antifungal activity through a dual-culture antagonism assay on PDA plates, according to the method described by [90].

#### 4.3.2. Detection of Diffusible Metabolite Production

For the detection of diffusible metabolite production, 100 µL of the KP32 strain culture was uniformly spread on PDA plates. After 24 h of incubation, a 5 mm agar–mycelium disc was placed at the center of the plate, separately for each pathogen. In the control, a 5 mm agar–mycelium disc was placed at the center of a plate inoculated with 100 µL of sterilized distilled water, instead of bacterial culture. Plates were then sealed with parafilm and incubated for 7–21 days (depending on the fungal strain) at 30 °C. The percent growth inhibition was calculated using the formula:

(1) $$\%\;\mathrm{Inhibition}=\frac{C\;-\;T}{C}\;\times \;100\%$$

where C is the radial growth (in mm) of the control fungal mycelium colony, and T is the radial growth (in mm) of the fungal mycelium growing in the presence of antagonist endophytic bacterial isolate. All treatments were performed in triplicate.

#### 4.3.3. Detection of Volatile Metabolites Production

For the detection of volatile metabolite production, 100 µL of bacterial culture was spread on the one half of a Petri dish containing LB medium, and a 5 mm agar disc of a pure culture of fungal phytopathogen was placed at the center of the other half of the Petri dish containing PDA medium, separately for each pathogen. Petri dishes inoculated only with fungi were used as controls. Plates were tightly sealed with parafilm and incubated at 30 °C for 7–21 days (depending on the fungal strain). The inhibition of fungal growth was measured using the formula presented above. The experiment was performed in triplicate.

#### 4.3.4. Evaluation of the Effect of KP32 Cell-Free Culture Filtrate

The KP32 strain was grown on Luria Bertani medium (LB Broth, BTL, Warsaw, Poland) in a 100 mL conical flask at 30 °C on a rotary shaker at 130 rpm. After 96 h of incubation, the culture was centrifuged at 10,000 rpm at 4 °C for 10 min, and cell-free culture filtrate was obtained by filtering the supernatant through a 0.22 µm pore size syringe filter. Next, the filtrate was thoroughly mixed with pre-cooled sterile PDA medium at a ratio of 1:10 (*v*/*v*), poured onto plates and allowed to solidify. A 5 mm-diameter agar–mycelium disc of the tested fungus was then placed at the center of the prepared PDA plates. Plugs of the tested fungi placed on non-amended (i.e., no cell-free culture filtrate added) PDA plates served as a control. Plates were incubated at 30 °C for 7–21 days (depending on the fungal strain). Mycelial growth inhibition (%) was measured as described above.

### 4.4. Genome Sequencing and Sequence Analysis

Genomic DNA of *S. quinivorans* KP32 was extracted using a GeneMatrix Bacterial and Yeast Genomic Purification Kit (EURx, Gdańsk, Poland). MicrobesNG performed the sequencing on an Illumina MiSeq platform with 2 × 250-bp paired-end reads. The results of the sequencing were subjected to a standard MicrobesNG analysis pipeline and were deposited in the GenBank database under the accession number JAHTKS000000000.1. Functional annotation of genes was performed using many tools and databases, including the eggNOG 5.0 orthology prediction pipeline (http:/eggnogdb.embl.de/, accessed on 26 February 2020) [91]. The genes assigned to multiple COG categories were counted as being present in each category. For gene function annotation, genes were compared to the KEGG database (http://www.genome.jp/kegg/, accessed on 26 February 2020) [92]. Functional and pathway analyses were also performed using the BlastKOALA web tool of KEGG (https://www.kegg.jp/blastkoala/, accessed on 26 February 2020) [92]. antiSMASH 5.0 was used for the identification of gene clusters responsible for the biosynthesis of secondary metabolites (https://antismash.secondarymetabolites.org, accessed on 17 November 2021) [93]. The CAZy database (http://www.cazy.org/, accessed on 15 June 2021) was used to classify cell wall-degrading enzymes (CWDEs) and divide them into different families. CAZy families were identified with dbCAN2, according to the DIAMOND database [94,95]. GIs were analyzed using the Island Viewer online tool version 4.0 (http://www.pathogenomics.sfu.ca/islandviewer/upload/, accessed on 17 November 2021) [96].

### 4.5. Phylogenetic Analysis

The phylogenetic tree was constructed based on the type strains and well-described strains belonging to the *Serratia* genus, with *Klebsiella pneumoniae* ATCC 13883T as an outgroup. The core proteomes of the 14 strains were determined and aligned using M1CR0B1AL1Z3R [97]. Poorly aligned regions were identified by Gblocks (version 0.91b) and removed (Integrated DNA Technologies, Iowa, IA, USA) [98], yielding 348,908 amino acids. A maximum-likelihood phylogenetic tree was obtained with MEGA 11 version 11.0.10 (Pennsylvania State University, Mueller Laboratory, University Park, PA, USA), with default settings and a 1000 bootstraps re-sampling [99].

### 4.6. Evaluation of the Effect of Fungal Pathogens on the Expression Level of Genes Engaged in Antifungal Activity

#### 4.6.1. Preparation of Fungal Filtrates

Fungal filtrates were prepared separately for each pathogen. The liquid medium was as described in Section 4.2, which was inoculated with plugs taken from actively growing fungal cultures and incubated for 21 days at 30 °C on a rotary shaker in darkness. The fungal biomass was collected by centrifugation (5000 rpm, 20 min, 4 °C), and the obtained supernatant was filtered through a 0.22 μm pore size syringe filter (Minisart Syringe Filter, Sartorius, Goettingen, Germany) to remove fungal cells and spores.

#### 4.6.2. Influence of Fungal Filtrates on Bacterial Genes Expression

A total of 25 mL of LB medium was inoculated with 1 mL of overnight LB culture of the KP32 strain, and 5 mL of fungal filtrate was added. The KP32 strain grown in LB medium without filtrate served as a control. Cultures were incubated with rotary shaking (130 rpm) at 30 °C for 96 h. After incubation, total RNA was isolated from the control and the filtrate-treated cultures using a GeneMATRIX Universal RNA Purification Kit (EURx, Gdańsk, Poland). The extracted RNA was additionally purified with TURBO DNA-free™ DNase (Invitrogen, ThermoFisher Scientific, Waltham, MA, USA), and the concentration and purity of obtained RNA were assessed using an ND-1000 NanoDrop spectrophotometer (ThermoFisher Scientific, Waltham, MA, USA). The synthesis of single-stranded cDNA was carried out in triplicate, using 1 µg of the total RNA, random hexamer primers and a RevertAid First Strand cDNA Synthesis Kit (ThermoFisher Scientific, Waltham, MA, USA). The generated cDNA was used as a template in qPCR reactions, performed using a LightCycler^®^ 480 Real-Time PCR System (Roche, Basel, Switzerland). The qPCR assay was performed in a 10 μL reaction volume with 5 µL LightCycler^®^ 480SYBR Green I Master (Roche, Basel, Switzerland), 2 µL PCR-grade water, 0.5 μM of each gene-specific primer, and 2 μL cDNA as a template. All PCR reactions were performed in LightCycler^®^480 Multiwell Plates 96 (Roche, Basel, Switzerland) under the following conditions: 10 min at 95 °C and 45 cycles of 15 s at 94 °C, 30 s at 60 °C, and 30 s at 72 °C. Three biological replicates and two technical replicates were used. The expression levels of the following genes were analyzed: *chiA*, *budA*, *hcnC*, *iucA*, *entB*, *pchB*, *katG*, *sodB*, and *mtlR*. The genes *gyrA* and *gyrB* were used as an internal controls, as commonly used standards in the study of gene expression of bacteria belonging to various genera, including those of *Serratia* [100,101,102]. The expression of internal genes was stable (CT = 15) across the treatments. In order to increase the stability of the measurements, two genes were used as an internal control. The primers used in this study were designed using Geneious Prime (version 2020.0.1; see Table 4). Three biological and two technical replicates were performed for each treatment. The amplification efficiency of primers for each gene were checked according to Taylor et al. [103] and primers with 90–110% reaction efficiency were used. The relative expression level was calculated according to Livak and Schmittgen [104].

### 4.7. Evaluation of the Effect of Fungal Pathogens on Lytic and Antioxidant Enzymes Activity

#### 4.7.1. Lytic Enzyme Activity

For the quantitative estimation of amylase, cellulase, protease, and chitinase activity, 200 mL of LB medium was inoculated with 5 mL culture of the KP32 strain, and 50 mL of the tested fungal filtrate was added. Fungal filtrates were prepared as described in Section 4.6.1. Bacteria grown in LB without filtrate served as a control. Cultures were incubated at 30 °C for 96 h and centrifuged (5000 rpm, 20 min, 4 °C). The cell-free supernatant was collected and used as an enzyme source. Amylase and cellulase activities were determined using the 3,5-dinitro salicylic acid (DNS) method, according to Ghose [105]. Protease activity was determined by the degradation of casein, according to Rais et al. [106]. Chitinase activity was measured according to the method described by Zarei et al. [107], with colloidal chitin as the substrate. The lytic enzyme activity was calculated following the formula described by Rais et al. [106].

#### 4.7.2. Antioxidant Enzyme Activity

For quantitative estimation of superoxide dismutase (SOD) and catalase (CAT) activities, 200 mL of LB broth was inoculated with 5 mL culture of the KP32 strain, and 50 mL of tested fungal filtrate was added (separately for each pathogen). Fungal filtrates were prepared as described in Section 4.6.1. Bacteria grown in LB medium without filtrate served as a control. The cultures were incubated at 30 °C for 96 h. Next, the bacterial cultures were centrifuged (5000 rpm, 20 min, 4 °C), and the obtained biomasses were suspended in 50 mM phosphate buffer (pH 7.2) and sonicated (20 kHz, 6× for 15 s) at 4 °C using a Vibra Cell (Sonics & Materials, Inc., Newtown, CT, USA). After centrifugation (15,000 rpm, 20 min, 4 °C), the cell-free extracts were used to measure the activity of SOD and CAT. The SOD activity was assayed spectrophotometrically at λ = 450 nm with the use of a SOD assay kit (Merck KGaA, Darmstadt, Germany), by reducing the tetrazolium salt, and calculated according to Zhang et al. [108]. The CAT activity was measured according to Banejree et al. [109]: at λ = 240 nm for 3 min as the decrease in the absorbance with H_2_O_2_ used as substrate (ɛ = 36,000 dm^3^mol^−1^cm^−1^). The protein concentration in the crude extract was calculated according to Bradford [110], with lysozyme as the standard. The specific activities of SOD and CAT are expressed as U mg^−1^ of protein.

### 4.8. Plant Growth Promotion Features of the KP32 Strain

The KP32 strain was examined for its ability to produce acetoin, 2,3-butanediol, IAA, SA, HCN, ACC deaminase, ammonia, and siderophores, as well as for phosphate solubilization ability, following the standard protocol [111,112,113,114,115] and Pikovskaya [116], respectively. Each analysis was completed in three biological repeats.

### 4.9. Colonization Features of the KP32 Strain

The KP32 strain was examined for different colonization features, including autoaggregation, biofilm formation, motility, and exopolysaccharide production, following the standard protocol [90,117,118]. The production of N-AHLs was evaluated qualitatively using the colorimetric method of Taghadosi et al. [119]. The presence of lactone compounds was indicated by a dark brown color, while the appearance of a yellow color indicated a lack of N-AHLs in the sample. Each analysis was completed in three biological repeats.

#### Utilization of Selected Organic Compounds as the Sole Source of Carbon and Energy

The ability of the isolated strain to grow in the presence of selected organic compounds as the sole source of carbon and energy was determined using a 96-well microplate. KP32 suspension with an optical density of about 0.250 at λ = 600 nm (OD_600_) was introduced into each well. The suspension was prepared as follows: the KP32 strain was cultivated in the LB medium for 24 h at 30 °C; after this, the culture was centrifuged (5000 rpm, 20 min, 4 °C), the supernatant was removed, and the bacterial biomass was centrifuged three times more (5000 rpm, 20 min, 4 °C), rinsing it after each centrifugation with sterile saline (0.9% NaCl) for purification from the remaining culture medium. After the final centrifugation, the bacterial biomass was suspended in mineral salt medium. Sufficient volumes of mineral salt medium and aqueous solution of aromatic compounds were also introduced into the wells. Growth of the isolated strain on microplates was tested in the presence of glucose, arabinose, rhamnose, mannose, trehalose, succinic acid, 4-hydroxyphenylacetic acid, fumaric acid, benzoic acid, mannitol, citric acid, and *p*-coumaric acid at concentrations of 1 mM [120]. At the same time, an abiotic culture was established, containing mineral salt medium and the tested aromatic compound, without a bacterial suspension. After inoculation, the microplate was incubated at 30 °C. After 24 h of incubation in the dark at 30 °C, the absorbance in each well was measured at a wavelength of 590 nm using a Biolog^®^ Microstation^TM^ (BIOLOG Inc., Hayward, CA, USA).

### 4.10. Statistical Analysis

The statistical analysis was carried out using the Microsoft Office Excel 2010 and Statistica 12.5 PL software (StatSoft^®^ Inc., Tulsa, OK, USA). Data are presented as the mean ± standard deviation (SD) of three biological replicates. For the analysis of the results of antagonistic tests and gene expression studies the independent Student’s *t*-test for the *p* < 0.05 was used. To compare activity of lytic and antioxidative enzymes data were analyzed using one-way ANOVA and evaluated by a post-hoc test of the means using the lowest significant differences (LSD) test (*p* < 0.05). Different letters indicate that the means differed significantly.

## 5. Conclusions

The newly isolated strain *Serratia quinivorans* KP32 presented biological activity against *Rhizoctonia solani* W70, *Colletotrichum dematium* K, *Fusarium avenaceum*, and *Sclerotinia sclerotiorum* K2291, inhibiting their growth to varying degrees. The ability of the KP32 strain to inhibit several phytopathogens may be conditioned by a combination of various biocontrol factors. Genome sequencing confirmed the presence of crucial genes encoding a wide range of mechanisms determining biological activity, plant growth promotion, and colonization. Studies of bacterial gene expression in response to exposure to fungal filtrates revealed alterations in the transcription of genes involved in the biosynthesis of chitinase, HCN, enterobactin, acetoin, and catalase. The KP32 strain showed the ability to produce a number of lytic enzymes (amylases, chitinases, and proteases) and antioxidant enzymes (CAT and SOD). The production of siderophores, VOCs, HCN, SA, and N-AHLs, as well as phosphate mobilization ability, indicates the possible involvement of various mechanisms in biocontrol of the selected phytopathogens. These mechanisms could be used in combination, leading to a synergistic inhibitory effect against phytopathogens. We also observed the active movement, self-aggregation, exopolysaccharide production, and biofilm formation abilities of the KP32 strain, which are features necessary for effective plant colonization. The biological activities of the KP32 strain and its ability to colonize plants strongly indicates the potential of *Serratia quinivorans* KP32 as an active biopesticide and plant growth-promoting agent.

## Figures and Tables

**Table 1:** General genome features of the KP32 strain.

Attribute	Value
Genome size (bp)	5,456,872
Contigs	107
G + C content (%)	64
Genes (total)	5194
CDSs (total)	5098
Genes (coding)	5024
Protein genes	5024
RNA genes	126
rRNAs	35
tRNAs	72
ncRNAs	12
Pseudogenes	44
Genes assigned to COGs	5018
Genes assigned to KEGG pathways	3342
BioProject ID	PRJNA743191
BioSample ID	SAMN20003760
GenBank accession number	JAHTKS000000000.1

**Table 2:** Activities of lytic and antioxidative enzymes measured for control KP32 and bacterial culture exposed to fungal filtrate.

Enzyme’s Activity	KP32	KP32 + RS	KP32 + FA	KP32 + CD	KP32 + SS
Protease (U mL^−1^)	10.32 ± 0.45 a	24.03 ± 0.21 b	9.05 ± 0.09 c	10.89 ± 0.10 d	4.98 ± 0.24 e
Amylase (U mL^−1^)	0.61 ± 0.25 a	0.64 ± 0.03 a	0.64 ± 0.01 a	0.21 ± 0.11 b	0.11 ± 0.14 c
Cellulase (U mL^−1^)	0.00 ± 0.00 a	0.00 ± 0.00 a	0.00 ± 0.00 a	0.00 ± 0.00 a	0.00 ± 0.00 a
Chitinase (U mL^−1^)	0.84 ± 0.12 a	0.85 ± 0.04 b	0.89 ± 0.21 a	2.12 ± 0.10 c	1.32 ± 05 d
Catalase (U mg^−1^ of protein)	2.84 ± 0.25 a	8.49 ± 0.07 b	4.05 ± 0.51 c	4.89 ± 0.10 c	2.47 ± 0.14 d
Superoxide dismutase (U mg^−1^ of protein)	0.43 ± 0.06 a	0.32 ± 0.09 a	0.60 ± 0.08 b	0.32 ± 0.11 c	0.24 ± 0.02 d

a,b,c,d,e indicate statistically significant differences, according to one-way ANOVA (*p* < 0.05) followed by the least significant differences (LSD) test.

**Table 3:** Functional characteristics of endophytic strain *S. quinivorans* KP32.

Features	Strain KP32
Plant growth promotion	
Acetoin and 2,3-butanediol production	+
IAA production (μg/mL)	14.32 ± 0.12
SA production (μg/mL)	5.43 ± 0.89
ACC deaminase production	+
Ammonia production	+
HCN production	+
Siderophore production	+
Phosphate solubilization (PSI)	2.75 ± 11
Colonization properties	
Autoaggregation (%)	
2 h	4.08 ± 0.21
24 h	34.2 ± 0.09
Biofilm formation (OD590 of crystal violet)	
24 h	0.297 ± 0.02
48 h	0.40 ± 0.12
72 h	0.52 ± 0.05
Motility (mm)	
Swimming (0.3%)	3.20 ± 0.3
Swarming (0.5%)	2.10 ± 0.07
Twitching (1%)	0.78 ± 0.15
Exopolysacharydes production	+
N-AHLs production	+

(+) capability, (−) lack of capability.

**Table 4:** Primer sequences (F: forward primer, R: reverse primer) used in RT-qPCR reaction.

Gene	Protein	Forward (5′-3’)	Reverse (5′-3’)	Tm (°C)	PCR Efficiency (%)	Slope	Product Size (bp)
*gyrA*	DNA gyrase subunit A	TGCGCTATATGCTGGTGGAC	GCAATTTTGGACATGCGCAC	53.8 51.8	97.24	−3.396	100
*gyrB*	DNA gyrase subunit B	CGGCGGCAAATTTGATGACA	AACCAGTTCCAGCTTCTCGG	51.8 53.8	99.00	−3.351	100
*chiA*	Chitinase	TGGAATGGCGATACCGGTAC	CCTTAAAGTTTGCCGTGCCC	53.8 53.8	101.35	−3.297	100
*budA*	Alpha-acetolactate decarboxylase	CGGTGTTTACGAAGGGGAGG	GAAGGCGATCAGTTCACCGT	55.9 53.8	90.94	−3.567	100
*hcnC*	Hydrogen cyanide synthase	ACAGCACTATCGACATGCCG	CCAGTCCAGCAGCGGATAAT	53.8 53.8	106.76	−3.178	100
*iucA*	Aerobactic synthase	GTATGCCCCGGAATACCAGG	CTGGGTCAGCGGATATGCTT	55.9 53.8	109.00	−3.116	100
*entB*	Enterobactin synthase	GATCAAGCAGGTGGTGGAGA	ATCGCTCTGCTGATTTGGCT	53.8 51.8	108.20	−3.147	100
*pchB*	Isochorismate pyruvate lyase	TCATTAAGCTGATCGCCCGG	ATGGCCTCAAAGCGCTCTTT	53.8 51.8	105.15	−3.202	100
*katG*	Catalase	GTTCACATTCCCAACTGCGC	ATCACCTTATTCCAGGCGGC	53.8 53.8	105.35	−3.201	100
*sodB*	Superoxide dismutase	CGGCGGCATCTTCAACAATG	GGCCAGTTTACCTTCAGGCT	53.8 53.8	104.53	−3.219	100
*mtlR*	Mannitol dehydrogenase	TCCCTTAAGTGAACGCCTCG	ATCGTGGCCAAACACCGTAT	51.8 53.8	110.28	−3.098	100

## Data Availability

Sequencing data and assembly are available at NCBI database under the BioProject accession number PRJNA743191. The data are also included in the Supplementary Materials available online, or will be available from the corresponding authors upon request.

## Supplementary Materials

The following supporting information can be downloaded at: https://www.mdpi.com/article/10.3390/ijms232415561/s1.

## Abbreviations

BCA	Biological Control Agents
ISR	Induced Systemic Resistance
N-AHLs	N-acyl-homoserine lactones
PCR	Polymerase Chain Reaction
VOCs	Volatile Organic Compounds
CDS	Protein-encoding sequences
KEGG	Kyoto Encyclopedia of Genes and Genomes
COG	Cluster of Orthologous Genes
NRPS	Nonribosomal Peptide-Synthetase
DHBA	Dihydroxybenzoic Acid
MFS	Major Facilitator Superfamily
IAA	Indole-3-acetic acid
TRP	Tryptophane
CBS	Cystathionine β synthase
CTH	Cystathionine-γ-lyase
GTS	Glutathione S-transferases
QS	Quorum sensing
CAZy	Carbohydrate-Active Enzymes
T2SS	Type II Secretion Systems
T6SS	Type VI Secretion Systems
T4PS	Type IV Pilus System
GI	Genomic Islands
RT-qPCR	quantitative reverse transcription PCR
SA	Salicylic Acid
ACC	1-aminocyclopropane-1-carboxylate
DF	Dworkin and Foster
HCN	Hydrogen Cyanide
SOD	Superoxide Dismutase
CAT	Catalase
EPS	Exopolysaccharide
CRA	Congo Red Agar
SEM	Scanning Electron Microscopy
PGI	Percent Growth Inhibition
LB	Luria-Bertani broth
CV	Crystal Violet
PSI	Phosphate Solubilizing Index
CAS	Chrome azurol S
PGPB	Plant Growth Promoting Bacteria
PGP	Plant Growth Promotion
PDA	Potato Dextrose Agar
CWDEs	Cell Wall-Degrading Enzymes
DNS	3,5-dinitro salicylic acid
LSD	Lowest Significant Difference

## References

[1] Marques-Pereira C., Proença D.N., Morais P.V. (2020). Genome sequences of *Serratia* strains revealed common genes in both serratomolides gene clusters. Biology.

[2] Matilla M.A., Nogellova V., Morel B., Krell T., Salmond G.P. (2016). Biosynthesis of the acetyl-CoA carboxylase-inhibiting antibiotic, andrimid in *Serratia* is regulated by Hfq and the LysR-type transcriptional regulator, AdmX. Environ. Microbiol..

[3] AL-Ghanem M.M. (2018). *Serratia* a novel source of secondary metabolites. Adv. Biotechnol. Microbiol..

[4] Niu H., Sun Y., Zhang Z., Zhao D., Wang N., Wang L., Guo H. (2022). The endophytic bacterial entomopathogen *Serratia marcescens* promotes plant growth and improves resistance against Nilaparvata lugens in rice. Microbiol. Res..

[5] Vaughan A.L., Altermann E., Glare T.R., Hurst M.R. (2022). Genome sequence of the entomopathogenic *Serratia entomophila* isolate 626 and characterisation of the species specific itaconate degradation pathway. BMC Genom..

[6] Soenens A., Imperial J. (2020). Biocontrol capabilities of the genus *Serratia*. Phytochem. Rev..

[7] Lahlali R., Ezrari S., Radouane N., Kenfaoui J., Esmaeel Q., El Hamss H., Belabess Z., Barka E.A. (2022). Biological control of plant pathogens: A global perspective. Microorganisms.

[8] Toffolatti S.L., Maffi D., Serrati L., Vercesi A. (2010). Histological and ultrastructural studies on the curative effects of mandipropamid on *Plasmopara viticola*. J. Phytopathol..

[9] Hazarika D.J., Goswami G., Gautom T., Parveen A., Das P., Barooah M., Chandra Boro R. (2019). Lipopeptide mediated biocontrol activity of endophytic *Bacillus subtilis* against fungal phytopathogens. BMC Microbiol..

[10] Wang M., Xing Y., Wang J., Xu Y., Wang G. (2014). The role of the *chi1* gene from the endophytic bacteria *Serratia proteamaculans* 336x in the biological control of wheat take-all. Can. J. Microbiol..

[11] Dhar Purkayastha G., Mangar P., Saha A., Saha D. (2018). Evaluation of the biocontrol efficacy of a *Serratia marcescens* strain indigenous to tea rhizosphere for the management of root rot disease in tea. PLoS ONE.

[12] Suryadi Y., Susilowati D.N., Fauziah F., Sayyed R. (2019). Management of Plant Diseases by PGPR-Mediated Induced Resistance with Special Reference to Tea and Rice Crops. Plant Growth Promoting Rhizobacteria for Sustainable Stress Management. Microorganisms for Sustainability.

[13] Kshetri L., Naseem F., Pandey P., Sayyed R. (2019). Role of *Serratia* sp. as Biocontrol Agent and Plant Growth Stimulator, with Prospects of Biotic Stress Management in Plant. Plant Growth Promoting Rhizobacteria for Sustainable Stress Management. Microorganisms for Sustainability.

[14] Su C., Xiang Z., Liu Y., Zhao X., Sun Y., Li Z., Li L., Chang F., Chen T., Wen X. (2016). Analysis of the genomic sequences and metabolites of *Serratia surfactantfaciens* sp. nov. YD25T that simultaneously produces prodigiosin and serrawettin W2. BMC Genom..

[15] Liu X., Jia J., Atkinson S., Cámara M., Gao K., Li H., Cao J. (2010). Biocontrol potential of an endophytic *Serratia* sp. G3 and its mode of action. World J. Microbiol. Biotechnol..

[16] Kamensky M., Ovadis M., Chet I., Chernin L. (2003). Soil-borne strain IC14 of *Serratia plymuthica* with multiple mechanisms of antifungal activity provides biocontrol of *Botrytis cinerea* and *Sclerotinia sclerotiorum* diseases. Soil Biol. Biochem..

[17] De Vleeschauwer D., Hö M. (2007). Using *Serratia plymuthica* to control fungal pathogens of plant. CAB Rev..

[18] Neupane S., Finlay R.D., Alström S., Elfstrand M., Högberg N. (2015). Transcriptional responses of the bacterial antagonist *Serratia plymuthica* to the fungal phytopathogen *Rhizoctonia solani*. Environ. Microbiol. Rep..

[19] Guitiérrez-Román M.I., Holguín-Meléndez F., Bello-Mendoza R., Guillén-Navarro K., Dunn M.F., Huerta-Palacios G. (2012). Production of prodigiosin and chitinases by tropical *Serratia marcescens* strains with potential to control plant pathogens. World J. Microbiol. Biotechnol..

[20] Ferraz H.G.M., Resende R.S., Moreira P.C., Silveira P.R., Milagres E.A., Oliveira J.R., Rodrigues F.A. (2015). Antagonistic rhizobacteria and jasmonic acid induce resistance against tomato bacterial spot. Plant Prot. Sci..

[21] Kumar A., Radhakrishnan E., Droby S., Singh V., Singh S., White J., Kumar A., Radhakrishnan E.K. (2019). Entry, colonization, and distribution of endophytic microorganisms in plants. Microbial Endophytes: Functional Biology and Applications.

[22] Nelkner J., Tejerizo G.T., Hassa J., Lin T.W., Witte J., Verwaaijen B., Winkler A., Bunk B., Spröer C., Overmann J. (2019). Genetic potential of the biocontrol agent *Pseudomonas brassicacearum* (formerly *P. trivialis*) 3Re2-7 unraveled by genome sequencing and mining, comparative genomics and transcriptomics. Genes.

[23] Li J., Yang Y., Dubern J.F., Li H., Halliday N., Chernin L., Gao K., Cámara M., Liu X. (2015). Regulation of GacA in *Pseudomonas chlororaphis* Strains Shows a Niche Specificity. PLoS ONE.

[24] Caneschi W.L., Sanchez A.B., Felestrino É.B., de Carvalho Lemes C.G., Cordeiro I.F., Fonseca N.P., Villa M.M., Vieira I.T., Moraes L.Â.G., de Almeida Barbosa Assis R. (2019). *Serratia liquefaciens* FG3 isolated from a metallophyte plant sheds light on the evolution and mechanisms of adaptive traits in extreme environments. Sci. Rep..

[25] Matteoli F.P., Passarelli-Araujo H., Reis R.J.A., da Rocha L.O., de Souza E.M., Aravind L., Olivares F.L., Venancio T.M. (2018). Genome sequencing and assessment of plant growth-promoting properties of a *Serratia marcescens* strain isolated from vermicompost. BMC Genom..

[26] Barnhart M.M., Chapman M.R. (2006). Curli biogenesis and function. Annu. Rev. Microbiol..

[27] Rezzonico F., Smits T.H., Duffy B. (2012). Detection of AI-2 receptors in genomes of *Enterobacteriaceae* suggests a role of type-2 quorum sensing in closed ecosystems. Sensors.

[28] Eida A.A., Bougouffa S., L’Haridon F., Alam I., Weisskopf L., Bajic V.B., Saad M.M., Hirt H. (2020). Genome Insights of the Plant-Growth Promoting Bacterium *Cronobacter muytjensii* JZ38 With Volatile-Mediated Antagonistic Activity Against *Phytophthora infestans*. Front. Microbiol..

[29] Andrés-Barrao C., Lafi F.F., Alam I., de Zélicourt A., Eida A.A., Bokhari A., Alzubaidy H., Bajic V.B., Hirt H., Saad M.M. (2017). Complete genome sequence analysis of *Enterobacter* sp. SA187, a plant multi-stress tolerance promoting endophytic bacterium. Front. Microbiol..

[30] Green E.R., Mecsas J. (2016). Bacterial Secretion Systems: An Overview. Microbiol. Spectr..

[31] Trunk K., Peltier J., Liu Y.C., Dill B.D., Walker L., Gow N.A.R., Stark M.J.R., Quinn J., Strahl H., Trost M. (2018). The type VI secretion system deploys antifungal effectors against microbial competitors. Nat. Microbiol..

[32] Wolska K., Jakubczak A. (2003). Wykrywanie biofilmu *Pseudomonas aeruginosa* na biomateriałach medycznych. Med. Doświadczalna Mikrobiol..

[33] Stepanovic S., Vukovic D., Hola V., Di Bonaventura G., Djukic S., Cirkovic I., Ruzicka F. (2007). Quantification of biofilm in microtiter plates: Overview of testing conditions and practical recommendations for assessment of biofilm production by staphylococci. APMIS.

[34] Sharma M., Saleh D., Charron J.-B., Jabaji S. (2020). A crosstalk between *Brachypodium* root exudates, organic acids, and *Bacillus velezensis* B26, a growth promoting bacterium. Front. Microbiol..

[35] He D.-C., He M.-H., Amalin D.M., Liu W., Alvindia D.G., Zhan J. (2021). Biological Control of Plant Diseases: An Evolutionary and Eco-Economic Consideration. Pathogens.

[36] Chandra H., Kumari P., Bisht R., Prasad R., Yadav S. (2020). Plant growth promoting *Pseudomonas aeruginosa* from *Valeriana wallichii* displays antagonistic potential against three phytopathogenic fungi. Mol. Biol. Rep..

[37] Afzal I., Shinwari Z.K., Sikandar S., Shahzad S. (2019). Plant beneficial endophytic bacteria: Mechanisms, diversity, host range and genetic determinants. Microbiol. Res..

[38] Shen S.S., Choi O.H., Lee S.M., Park C.S. (2002). In vitro and in vivo activities of biocontrol agent, *Serratia plymuthica* A21-4, against *Phytophthora capsici*. Plant Pathol. J..

[39] Frankowski J., Lorito M., Scala F., Schmid R., Berg G., Bahl H. (2001). Purification and properties of two chitinolytic enzymes of *Serratia plymuthica* HRO-C48. Arch. Microbiol..

[40] Roberts D., Lohrke S., Meyer S., Buyer J., Bowers J., Baker C., Li W., de Souza J., Lewis J., Chung S. (2005). Biocontrol agents applied individually and in combination for suppression of soilborne diseases of cucumber. Crop Prot..

[41] Parani K., Shetty G.P., Saha B.K. (2011). Isolation of *Serratia marcescens* SR1 as a source of chitinase having potentiality of using as a biocontrol agent. Indian J. Microbiol..

[42] Chakraborty U., Chakraborty B.N., Chakraborty A.P. (2010). Influence of *Serratia marcescens* TRS-1 on growth promotion and induction of resistance in *Camellia sinensis* against *Fomes lamaoensis*. J. Plant Interact..

[43] Varma A., Bakshi M., Lou B., Hartmann A., Oelmueller R. (2012). *Piriformospora indica*: A Novel Plant Growth Promoting Mycorrhizal Fungus. Agric. Res..

[44] Dashti N., Prithiviraj B., Hynes R.K., Smith D.L. (2000). Root and Rhizosphere Colonization of Soybean (*Glycine max* (L.) Merr.) by Plant-Growth-Promoting Rhizobacteria at Low Root Zone Temperatures and under Short-Season Conditions. J. Agron. Crop Sci..

[45] Berg G., Roskot N., Steidle A., Eberl L., Zock A., Smalla K. (2002). Plant-dependent genotypic and phenotypic diversity of antagonistic rhizobacteria isolated from different *Verticillium* host plants. Appl. Environ. Microbiol..

[46] Zheng A., Lin R., Zhang D., Qin P., Xu L., Ai P., Ding L., Wang Y., Chen Y., Liu Y. (2013). The evolution and pathogenic mechanisms of the rice sheath blight pathogen. Nat. Commun..

[47] Marzouk T., Chaouachi M., Sharma A., Jallouli S., Mhamdi R., Kaushik N., Djébali N. (2021). Biocontrol of *Rhizoctonia solani* using volatile organic compounds of solanaceae seed-borne endophytic bacteria. Postharvest Biol. Technol..

[48] Gkarmiri K., Finlay R.D., Alström S., Thomas E., Cubeta M.A., Högberg N. (2015). Transcriptomic changes in the plant pathogenic fungus *Rhizoctonia solani* AG-3 in response to the antagonistic bacteria *Serratia proteamaculans* and *Serratia plymuthica*. BMC Genom..

[49] Li P., Kwok A.H.Y., Jiang J., Ran T., Xu D., Wang W., Leung F.C. (2015). Comparative Genome Analyses of *Serratia marcescens* FS14 Reveals Its High Antagonistic Potential. PLoS ONE.

[50] Delgado N., Olivera M., Cádiz F., Bravo G., Montenegro I., Madrid A., Fuentealba C., Pedreschi R., Salgado E., Besoain X. (2021). Volatile Organic Compounds (VOCs) Produced by *Gluconobacter cerinus* and *Hanseniaspora osmophila* Displaying Control Effect against Table Grape-Rot Pathogens. Antibiotics.

[51] Weise T., Thurmer A., Brady S., Kai M., Daniel R., Gottschalk G., Piechulla B. (2014). VOC emission of various *Serratia* species and isolates and genome analysis of *Serratia plymuthica* 4Rx13. FEMS Microbiol. Lett..

[52] Nascimento F., Vicente C., Cock P., Tavares M., Rossi M., Hasegawa K., Mota M. (2018). From plants to nematodes: *Serratia grimesii* BXF1 genome reveals an adaptation to the modulation of multi-species interactions. Microb. Genom..

[53] Yi H.S., Ahn Y.R., Song G.C., Ghim S.Y., Lee S., Lee G., Ryu C.M. (2016). Impact of a Bacterial Volatile 2,3-Butanediol on *Bacillus subtilis* Rhizosphere Robustness. Front. Microbiol..

[54] van der Lelie D., Taghavi S., Monchy S., Schwender J., Miller L., Ferrieri R., Rogers A., Wu X., Zhu W., Weyens N. (2009). Poplar and its Bacterial Endophytes: Coexistence and Harmony. Crit. Rev. Plant Sci..

[55] Taghavi S., Garafola C., Monchy S., Newman L., Hoffman A., Weyens N., Barac T., Vangronsveld J., van der Lelie D. (2009). Genome survey and characterization of endophytic bacteria exhibiting a beneficial effect on growth and development of poplar trees. Appl. Environ. Microbiol..

[56] Umesha S., Singh K.P.P., Singh R., Lakhan Singh R., Mondal S. (2018). Microbial biotechnology and sustainable agriculture. Biotechnology for Sustainable Agriculture.

[57] Peng G., Zhao X., Li Y., Wang R., Huang Y., Qi G. (2019). Engineering *Bacillus velezensis* with high production of acetoin primes strong induced systemic resistance in *Arabidopsis thaliana*. Microbiol. Res..

[58] Fu L.H., Hu K.D., Hu L.Y., Li Y.H., Hu L.B., Yan H., Liu Y.S., Zhang H. (2014). An antifungal role of hydrogen sulfide on the postharvest pathogens *Aspergillus niger* and *Penicillium italicum*. PLoS ONE.

[59] Popova A.A., Koksharova O.A., Lipasova V.A., Zaitseva J.V., Katkova-Zhukotskaya O.A., Eremina S.I., Mironov A.S., Chernin L.S., Khmel I.A. (2014). Inhibitory and toxic effects of volatiles emitted by strains of *Pseudomonas* and *Serratia* on growth and survival of selected microorganisms, *Caenorhabditis elegans*, and *Drosophila melanogaster*. Biomed. Res. Int..

[60] Etminani F., Harighi B. (2018). Isolation and Identification of Endophytic Bacteria with Plant Growth Promoting Activity and Biocontrol Potential from Wild Pistachio Trees. Plant Pathol. J..

[61] Dorjey S., Dolkar D., Sharma R. (2017). Plant growth promoting rhizobacteria *Pseudomonas*: A review. Int. J. Curr. Microbiol..

[62] Purushotham P., Arun P.V., Prakash J.S., Podile A.R. (2012). Chitin binding proteins act synergistically with chitinases in *Serratia proteamaculans* 568. PLoS ONE.

[63] Compant S., Duffy B., Nowak J., Clément C., Barka E.A. (2005). Use of plant growth promoting bacteria for biocontrol of plant diseases: Principles, mechanisms of action, and future prospects. Appl. Environ. Microbiol..

[64] Someya N., Nakajima M., Watanabe K., Hibi T., Akutsu K. (2005). Potential of *Serratia marcescens* strain B2 for biological control of rice sheath blight. Biocontrol Sci. Technol..

[65] Ben Slama H., Triki M.A., Bouket A.C., Mefteh B.F., Alenezi F.N., Luptakova L., Cherif-Silini H., Vallat A., Oszako T., Gharsallah N. (2019). Screening of the High Rhizosphere Competent *Limoniastrum monopetalum’* Culturable Endophyte Microbiota Allows the Recovery of Multifaceted and Versatile Biocontrol Agents. Microorganisms.

[66] Kumar A., Vandana R.S., Singh M., Pandey K.D. (2019). Plant growth promoting rhizobacteria (PGPR). A promising approach to disease management. Microbes and Environmental Management.

[67] Faltin F., Lottmann J., Grosch R., Berg G. (2004). Strategy to select and assess antagonistic bacteria for biological control of *Rhizoctonia solani* Kühn. Can. J. Microbiol..

[68] Shanmugaiah V., Nithya K., Harikrishnan H., Jayaprakashvel M., Balasubramanian N., Kannan V.R., Bastas K.K. (2015). Biocontrol mechanisms of siderophores against bacterial plant pathogens. Sustainable Approaches to Controlling Plant Pathogenic Bacteria.

[69] Ahmed E., Holmström S.J. (2014). Siderophores in environmental research: Roles and applications. Microbial. Biotechnol..

[70] Schiessl K.T., Janssen E.M., Kraemer S.M., McNeill K., Ackermann M. (2017). Magnitude and Mechanism of Siderophore-Mediated Competition at Low Iron Solubility in the *Pseudomonas aeruginosa* Pyochelin System. Front. Microbiol..

[71] Bigirimana J., Höfte M. (2002). Induction of systemic resistance to *Colletotrichum lindemuthianum* in bean by a benzothiadiazole derivative and rhizobacteria. Phytoparasitica.

[72] Sharma A., Johri B.N. (2003). Growth promoting influence of siderophore producing *Pseudomonas strains* GRP3A and PRS9 in maize (*Zea mays* L.) under iron limiting conditions. Microbiol. Res..

[73] Press C.M., Loper J.E., Kloepper J.W. (2001). Role of iron in rhizobacteria mediated induced systemic resistance of cucumber. Phytopathology.

[74] Singh D.P., Gupta V.K., Prabha R. (2019). Microbial Interventions in Agriculture and Environment: Rhizosphere, Microbiome and Agro-Ecology.

[75] Saha R., Saha N., Donofrio R.S., Bestervelt L.L. (2013). Microbial siderophores: A mini review. J. Basic Microbiol..

[76] Bargaz A., Lyamlouli K., Chtouki M., Zeroual Y., Dhiba D. (2018). Soil Microbial Resources for Improving Fertilizers Efficiency in an Integrated Plant Nutrient Management System. Front. Microbiol..

[77] Sharma S.B., Sayyed R.Z., Trivedi M.H., Gobi T.A. (2013). Phosphate solubilizing microbes: Sustainable approach for managing phosphorus deficiency in agricultural soils. Springerplus.

[78] Anzuay M.S., Ludueña L.M., Angelini J.G., Fabra A., Taurian T. (2015). Beneficial effects of native phosphate solubilizing bacteria on peanut (*Arachis hypogaea* L.) growth and phosphorus acquisition. Symbiosis.

[79] Dipak P., Sankar S. (2017). Isolation and characterization of phosphate solubilizing bacterium *Pseudomonas aeruginosa* KUPSB12 with antibacterial potential from river Ganga, India. Ann. Agrar. Sci..

[80] Zeng Q., Wu X., Wang J., Ding X. (2017). Phosphate solubilization and gene expression of phosphate-solubilizing bacterium *Burkholderia multivorans* WS-FJ9 under different levels of soluble phosphate. J. Microbiol. Biotechnol..

[81] Pande A., Pandey P., Mehra S., Singh M., Kaushik S. (2017). Phenotypic and genotypic characterization of phosphate solubilizing bacteria and their efficiency on the growth of maize. J. Genet. Eng. Biotechnol..

[82] Zhang S., Moyne A.L., Reddy M.S., Kloepper J.W. (2002). The role of salicylic acid in induced systemic resistance elicited by plant growth-promoting rhizobacteria against blue mold of tobacco. Biol. Control.

[83] Dubuis C., Keel C., Haas D. (2007). Dialogues of root-colonizing biocontrol pseudomonads. Eur. J. Plant Pathol..

[84] Raaijmakers J.M., Paulitz T.C., Steinberg C., Alabouvette C., Moënne-Loccoz Y. (2009). The rhizosphere: A playground and battlefeld for soilborne pathogens and benefcial microorganisms. Plant Soil.

[85] Danese P.N., Pratt L.A., Kolter R. (2000). Exopolysaccharide production is required for development of *Escherichia coli* K-12 biofilm architecture. J. Bacteriol..

[86] Zhu M.-L., Wu X.-Q., Wang Y.-H., Dai Y. (2020). Role of Biofilm Formation by *Bacillus pumilus* HR10 in Biocontrol against Pine Seedling Damping-Off Disease Caused by *Rhizoctonia solani*. Forests.

[87] Shehata H.R., Ettinger C.L., Eisen J.A., Raizada M.N. (2016). Genes Required for the Anti-fungal Activity of a Bacterial Endophyte Isolated from a Corn Landrace Grown Continuously by Subsistence Farmers Since 1000 BC. Front. Microbiol..

[88] Kandel S.L., Joubert P.M., Doty S.L. (2017). Bacterial Endophyte Colonization and Distribution within Plants. Microorganisms.

[89] Hover T., Maya T., Ron S., Sandovsky H., Shadkchan Y., Kijner N., Mitiagin Y., Fichtman B., Harel A., Shanks R.M. (2016). Mechanisms of Bacterial (*Serratia marcescens*) Attachment to, Migration along, and Killing of Fungal Hyphae. Appl. Environ. Microbiol..

[90] Chlebek D., Pinski A., Żur J., Michalska J., Hupert-Kocurek K. (2020). Genome Mining and Evaluation of the Biocontrol Potential of *Pseudomonas fluorescens* BRZ63, a New Endophyte of Oilseed Rape (*Brassica napus* L.) against Fungal Pathogens. Int. J. Mol. Sci..

[91] Huerta-Cepas J., Szklarczyk D., Heller D., Hernández-Plaza A., Forslund S.K., Cook H., Mende D.R., Letunic I., Rattei T., Jensen L.J. (2019). EggNOG 5.0: A hierarchical, functionally and phylogenetically annotated orthology resource based on 5090 organisms and 2502 viruses. Nucleic Acids Res..

[92] Kanehisa M., Sato Y., Kawashima M., Furumichi M., Tanabe M. (2016). KEGG as a reference resource for gene and protein annotation. Nucleic Acids Res..

[93] Blin K., Shaw S., Steinke K., Villebro R., Ziemert N., Lee S.Y., Medema M.H., Weber T. (2019). AntiSMASH 5.0: Updates to the secondary metabolite genome mining pipeline. Nucleic Acids Res..

[94] Yin Y., Mao X., Yang J., Chen X., Mao F., Xu Y. (2012). dbCAN: A web resource for automated carbohydrate-active enzyme annotation. Nucleic Acids Res..

[95] Zhang H., Yohe T., Huang L., Entwistle S., Wu P., Yang Z., Busk P.K., Xu Y., Yin Y. (2018). dbCAN2: A meta server for automated carbohydrate-active enzyme annotation. Nucleic Acids Res..

[96] Bertelli C., Laird M.R., Williams K.P., Lau B.Y., Hoad G., Winsor G.L., Brinkman F.S.L., Simon Fraser University Research Computing Group (2017). IslandViewer 4: Expanded prediction of genomic islands for larger-scale datasets. Nucleic Acids Res..

[97] Avram O., Rapoport D., Portugez S., Pupko T. (2019). M1CR0B1AL1Z3R—A user-friendly web server for the analysis of large-scale microbial genomics data. Nucleic Acids Res..

[98] Talavera G., Castresana J. (2007). Improvement of phylogenies after removing divergent and ambiguously aligned blocks from protein sequence alignments. Syst. Biol..

[99] Kumar S., Stecher G., Li M., Knyaz C., Tamura K. (2018). MEGA X: Molecular evolutionary genetics analysis across computing platforms. Mol. Biol. Evol..

[100] Vicente C.S.L., Nascimento F.X., Ikuyo Y., Cock P.J., Mota M., Hasegawa K. (2016). The genome and genetics of a high oxidative stress tolerant *Serratia* sp. LCN16 isolated from the plant parasitic nematode *Bursaphelenchus xylophilus*. BMC Genom..

[101] Zur J., Pinski A., Wojcieszynska D., Smułek W., Guzik U. (2020). Diclofenac degradation—Enzymes, genetic background and cellular alterations triggered in diclofenac-metabolizing strain *Pseudomonas moorei* KB4. Int. J. Mol. Sci..

[102] Dąbrowska G.B., Tylman-Mojżeszek W., Mierek-Adamska A., Richert A., Hrynkiewicz K. (2021). Potential of Serratia plymuthica IV-11-34 strain for biodegradation of polylactide and poly(ethylene terephthalate). Int. J. Biol. Macromol..

[103] Taylor S., Wakem M., Dijkman G., Alsarraj M., Nguyen M. (2010). A practical approach to RT-qPCR-Publishing data that conform to the MIQE guidelines. Methods.

[104] Livak K.J., Schmittgen T.D. (2001). Analysis of relative gene expression data using real-time quantitative PCR and the 2^−∆∆CT^ method. Methods.

[105] Ghose T.K. (1987). Measurement of cellulase activities. Pure Appl. Chem..

[106] Rais A., Jabeen Z., Shair F., Hafeez F.Y., Hassan M.N. (2017). *Bacillus* spp., a bio-control agent enhances the activity of antioxidant defense enzymes in rice against *Pyricularia oryzae*. PLoS ONE.

[107] Zarei M., Aminzadeh S., Zolgharnein H., Safahieh A., Daliri M., Noghabi K.A., Ghoroghi A., Motallebi A. (2011). Characterization of a chitinase with antifungal activity from a native *Serratia marcescens* B4A. Braz. J. Microbiol..

[108] Zhang C., Bruins M.E., Yang Z.Q., Liu S.T., Rao P.F. (2016). A new formula to calculate activity of superoxide dismutase in indirect assays. Anal. Biochem..

[109] Banerjee G., Pandey S., Ray A.K., Kumar R. (2015). Bioremediation of heavy metals by a novel bacterial strain *Enterobacter cloaca* and its antioxidant enzyme activity, flocculant production and protein expression in presence of lead, cadmium and nickel. Water Air Soil Pollut..

[110] Bradford M.M. (1976). A rapid and sensitive method for the quantitation of microgram quantities of proteins utilizing the principle of protein-dye binding. Anal. Biochem..

[111] Johnston-Monje D., Raizada M.N. (2013). Conservation and diversity of seed associated endophytes in *Zea* across boundaries of evolution, ethnography and ecology. PLoS ONE.

[112] Syamala M., Sivaji M. (2017). Functional characterization of various plant growth promoting activity of *Pseudomonas fluorescens* and *Bacillus subtilis* from *Aloe vera* rhizosphere. J. Pharmacogn. Phytochem..

[113] Sandhya V., Shrivastava M., Ali S.Z., Prasad V.S.S.K. (2017). Endophytes from maize with plant growth promotion and biocontrol activity under drought stress. Russ. Agric. Sci..

[114] Cappuccino J.G., Sherman N. (1992). Biochemical Activities of Microorganisms. Microbiology, A Laboratory Manual.

[115] Schwyn B., Neilands J.B. (1987). Universal chemical assay for the detection and determination of siderophores. Anal. Biochem..

[116] Pikovskaya R.I. (1948). Mobilization of phosphorus in soil in connection with vital activity of some microbial species. Microbiology.

[117] Naveed M., Mitter B., Yousaf S., Pastar M., Afzal M., Sessitsch A. (2014). The endophyte *Enterobacter* sp. FD17: A maize growth enhancer selected based on rigorous testing of plant beneficial traits and colonization characteristics. Biol. Fertil. Soils.

[118] Freeman D.J., Falkiner F.R., Keane C.T. (1989). New method for detecting slime production by coagulase negative staphylococci. J. Clin. Pathol..

[119] Taghadosi R., Shakibaie M.R., Masoumi S. (2015). Biochemical detection of N-Acyl homoserine lactone from biofilm-forming uropathogenic *Escherichia coli* isolated from urinary tract infection samples. Rep. Biochem. Mol. Biol..

[120] Michalska J., Piński A., Żur J., Mrozik A. (2020). Selecting Bacteria Candidates for the Bioaugmentation of Activated Sludge to Improve the Aerobic Treatment of Landfill Leachate. Water.

