# Supplementary material for: Genetic Determinants of Antagonistic Interactions and the Response of New Endophytic Strain Serratia quinivorans KP32 to Fungal Phytopathogens
Source: Int J Mol Sci. 2022 Dec 8;23(24):15561. doi: 10.3390/ijms232415561 (PMC9779691; doi:10.3390/ijms232415561)
Supplement: Supplementary file 1 [file ijms-23-15561-s001.zip › ijms-2028999-supplementary.pdf]

Supplementary materials: **Genetic determinants of antagonistic interactions and response of new endophytic strain *Serratia quinivorans* KP32 to fungal phytopathogens.**

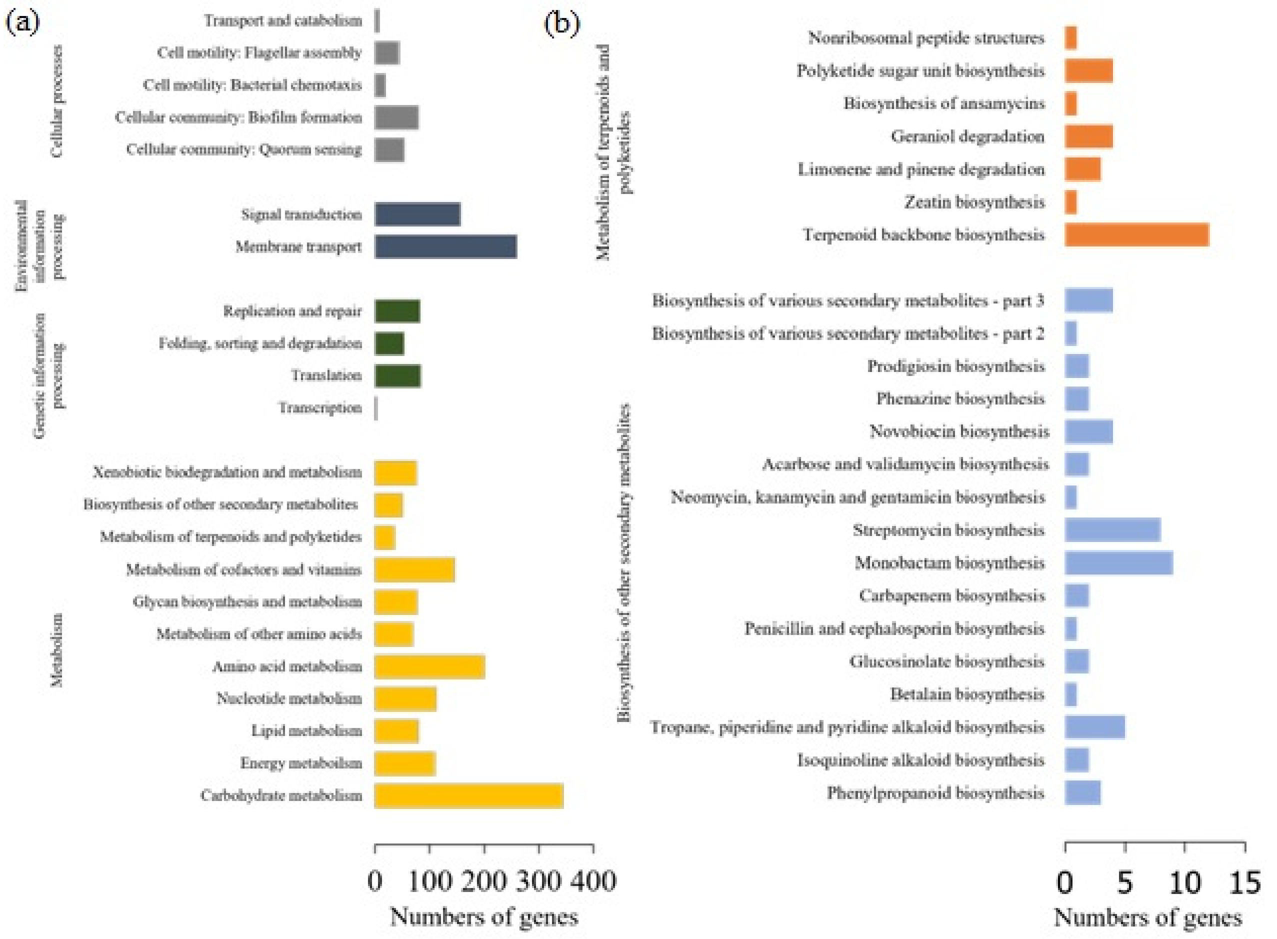

Figure S1. KEGG pathway classification of predicted genes in the KP32 strain (**a**), number of predicted genes involved in biosynthesis of cluster secondary metabolites and metabolism of terpenoids and polyketides (**b**).

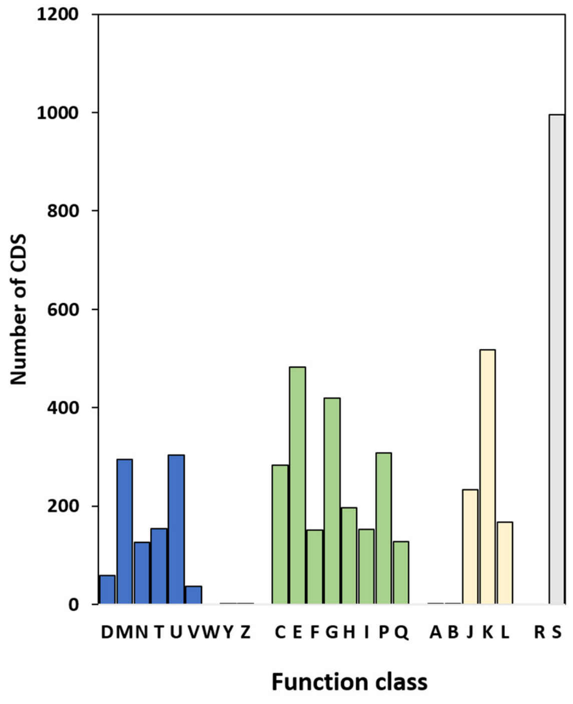

COG	Description	KP32 CDS	KP32 CDS (%)	Funcional group
D	Cell cycle control. cell division, chromosome partitioning	59	1,18	Cellular process and signaling
M	Cell wall/membrane/envelope biogenesis	295	5,88	
N	Cell motility	126	2,51	
T	Signal transduction mechanisms	154	3,07	
U	Intracellular trafficking, secretion, and vesicular transport	304	6,06	
V	Defense mechanisms	37	0,74	
W	Extracellular structures	-	-	
Y	Nuclear structure	1	0,02	
Z	Cytoskeleton	1	0,02	
C	Energy production and conversion	284	5,66	Metabolism
E	Amino acid transport and metabolism	483	9,63	
F	Nucleotide transport and metabolism	152	3,03	
G	Carbohydrate transport and metabolism	420	8,37	
H	Coenzyme transport and metabolism	197	3,93	
I	Lipid transport and metabolism	153	3,05	
P	Inorganic ion transport and metabolism	308	6,14	
Q	Secondary metabolites biosynthesis, transport and catabolism	128	2,55	
A	RNA processing and modification	1	0,02	Information storage and processing
B	Chromatin structure and dynamics	1	0,02	
J	Translation. ribosomal structure and biogenesis	234	4,66	
K	Transcription	518	10,32	
L	Replication. recombination and repair	167	3,33	
R	General function prediction only	-	-	Poorly charact erized
S	Function unknown	995	19,83	

Figure S2. Functional COG classification of predicted genes in the KP32 strain. Colored bars indicate the CDS assigned to each COG category.

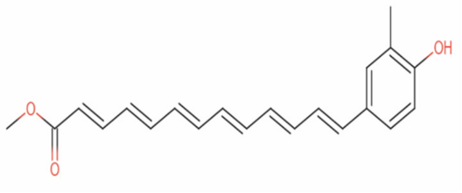

Arylpolyene
$C_{21}H_{22}O_3$

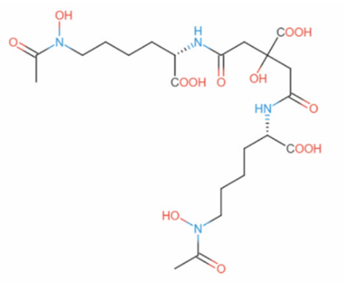

Aerobactin
$C_{22}H_{36}N_4O_{13}$

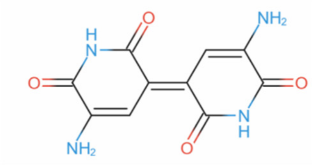

Indiogine
$C_{10}H_8N_4O_4$

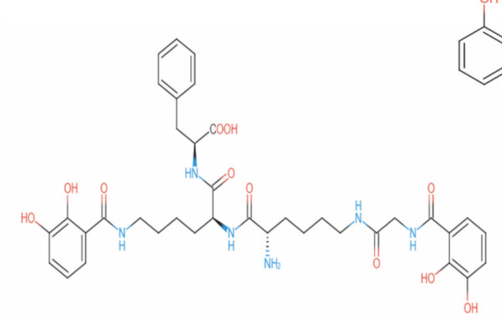

Amonabactin P 750
$C_{37}H_{46}N_6O_{11}$

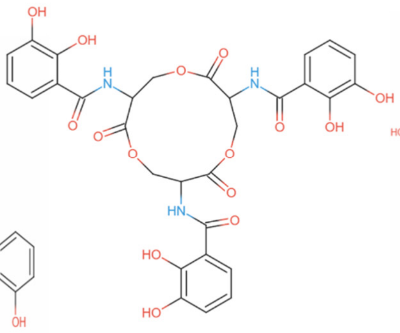

Enterobactin
$C_{30}H_{27}N_3O_{15}$

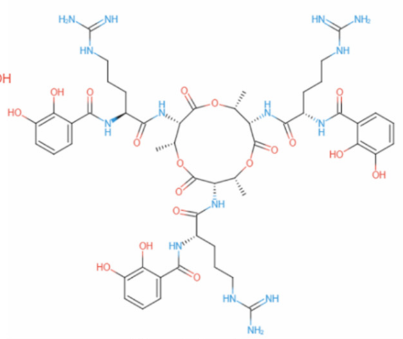

Streptobactin
$C_{51}H_{69}N_{15}O_{18}$

Figure S3. Predicted structures of secondary metabolites recognized for the KP32 strain using PubChem.

Table S1. Secondary metabolite gene clusters identified in the *S. quinivorans* KP32 strain using antiSMASH v. 5.1.2.

Region	Number of genes	Type	From	To	Most similar known cluster	Similarity
Region 2.1	56	NRPS	22.349	74.211		
Region 2.2	45	Siderophore, Aryl polyene	113.598	155.455	Arylpolyene	77%
					Aerobactin	77%
Region 5.1	23	Betalactone	46.504	72.176		
Region 11.1	34	NRPS-like, thiopeptide	630.792	674.628	Indiogine	NRP, 80%
Region 15.1	24	Nukleoside	3.493	25.905		
Region 31.1	45	NRPS	265.207	318.809	Amonabactin P 750	NRP, 57%
					Enterobactin	20%
					Streptobactin	24%
Region 34.1	40	NRPS-like	56.898	99.879		

NRP - *Nonribosomal* peptides, NRPS - *Nonribosomal* peptide synthetase

Table S2. Genes potentially involved in biocontrol, PGP and colonization in the KP32 genome.

Gene	Accession number	KO	Gene product	Activity
entA	MBV6692457.1	K00216	2,3-dihydro-2,3-dihydroxybenzoate dehydrogenase [EC:1.3.1.28]	***Biosynthesis and transport of siderophores***
entB	MBV6692458.1	K01252	bifunctional isochorismate lyase / aryl carrier protein [EC:3.3.2.1 6.3.2.14]	
entE	MBV6692459.1	K02363	2,3-dihydroxybenzoate-AMP ligase [EC:6.3.2.14 2.7.7.58]	
entC;	MBV6692461.1	K02361	isochorismate synthase [EC:5.4.4.2]	
fepB;	MBV6692462.1	K23185	ferric enterobactin transport system substrate-binding protein	
entS	MBV6692463.1	K08225	MFS transporter, ENTS family, enterobactin (siderophore) exporter	
fepD	MBV6692464.1	K23186	ferric enterobactin transport system permease protein	
fepG	MBV6692465.1	K23187	ferric enterobactin transport system permease protein	
fepC	MBV6692466.1	K23188	ferric enterobactin transport system ATP-binding protein [EC:7.2.2.17]	
entF	MBV6692467.1	K02364	enterobactin synthetase component F [EC:6.3.2.14]	
fes	MBV6692469.1	K07214	iron(III)-enterobactin esterase [EC:3.1.1.108]	
fepA	MBV6692470.1	K19611	pfeA, iroN, pirA; ferric enterobactin receptor	
entD	MBV6692784.1	K02362	enterobactin synthetase component D [EC:6.3.2.14 2.7.8.-]	
fhuC	MBV6694398.1	K10829	ferric hydroxamate transport system ATP-binding protein [EC:7.2.2.16]	
fhuD	MBV6694399.1	K23227	ferric hydroxamate transport system substrate-binding protein	
fhuB	MBV6694400.1	K23228	ferric hydroxamate transport system permease protein	
iutA	MBV6693781.1	K02014	iron complex outermembrane recepter protein	
feoA	MBV6695302.1	K04758	ferrous iron transport protein A	
feoB	MBV6695303.1	K04759	ferrous iron transport protein B	
feoC	MBV6695304.1	K07490	ferrous iron transport protein C	
efeB	MBV6691879.1	K16301	deferrochelatase/peroxidase EfeB [EC:1.11.1.-]	
efeO	MBV6691880.1	K07224	iron uptake system component EfeO	
efeU	MBV6691881.1	K07243	high-affinity iron transporter	
iucA	MBV6693894.1	K03894	N2-citryl-N6-acetyl-N6-hydroxylysine synthase [EC:6.3.2.38]	
iucB	MBV6693895.1	K03896	acetyl CoA:N6-hydroxylysine acetyl transferase [EC:2.3.1.102]	
iucC	MBV6693896.1	K03895	aerobactin synthase [EC:6.3.2.39]	
iucD	MBV6693897.1	K03897	lysine N6-hydroxylase [EC:1.14.13.59]	
dps	MBV6690874.1	K04047	starvation-inducible DNA-binding protein	
sufD	MBV6694156.1	K09015	Fe-S cluster assembly protein SufD	
sufC	MBV6694157.1	K09013	Fe-S cluster assembly ATP-binding protein	
sufB	MBV6694158.1	K09014	Fe-S cluster assembly protein SufB	
sufA	MBV6694159.1	K05997	Fe-S cluster assembly protein SufA	
sufE	MBV6693317.1	K02426	cysteine desulfuration protein SufE	
pstS	MBV6693473.1	K02040	phosphate transport system substrate-binding protein	***Phosphate metabolism***
pstC	MBV6693474.1	K02037	phosphate transport system permease protein	
pstA	MBV6693475.1	K02038	phosphate transport system permease protein	
pstB	MBV6693476.1	K02036	phosphate transport system ATP-binding protein [EC:7.3.2.1]	
phoU	MBV6693477.1	K02039	phosphate transport system protein	
phnA	MBV6690599.1	K06193	protein PhnA	
ppk2	MBV6691499.1	K22468	polyphosphate kinase [EC:2.7.4.34]	
ppa	MBV6695132.1	K01507	inorganic pyrophosphatase [EC:3.6.1.1]	
phoA	MBV6690920.1	K01077	alkaline phosphatase [EC:3.1.3.1]	
phoB	MBV6695340.1	K07657	phosphate regulon transcriptional regulatory protein PhoB	
phoR	MBV6695341.1	K07636	phosphate regulon transcriptional regulatory protein PhoR	
phoH	MBV6690598.1	K06217	phosphate starvation-inducible protein PhoH and related proteins	
gcd	MBV6692846.1	K00117	quinoprotein glucose dehydrogenase [EC:1.1.5.2]	
ipdC	MBV6693804.1	K04103	indolepyruvate decarboxylase [EC:4.1.1.74]	***IAA biosynthesis***
aspC	MBV6691124.1	K00813	aspartate aminotransferase [EC:2.6.1.1]	
aldA	MBV6691460.1	K07248	lactaldehyde dehydrogenase / glycolaldehyde dehydrogenase [EC:1.2.1.22 1.2.1.21]	
trpE	MBV6691605.1	K01657	anthranilate synthase component I [EC:4.1.3.27]	***Tryptophan biosynthesis***
trpG	MBV6691606.1	K01658	anthranilate synthase component II [EC:4.1.3.27]	

trpD	MBV6691607.1	K00766	anthranilate phosphoribosyltransferase [EC:2.4.2.18]	
trpCF	MBV6691608.1	K13498	indole-3-glycerol phosphate synthase / phosphoribosylanthranilate isomerase [EC:4.1.1.48 5.3.1.24]	
trpB	MBV6691609.1	K01696	tryptophan synthase beta chain [EC:4.2.1.20]	
trpA	MBV6691610.1	K01695	tryptophan synthase alpha chain [EC:4.2.1.20]	
speA	MBV6694404.1	K01585	arginine decarboxylase [EC:4.1.1.19]	***Spermidine biosynthesis***
speB	MBV6694405.1	K01480	agmatinase [EC:3.5.3.11]	
speD	MBV6694374.1	K01611	AMD1; S-adenosylmethionine decarboxylase [EC:4.1.1.50]	
speE	MBV6694375.1	K00797	spermidine synthase [EC:2.5.1.16]	
cysC	MBV6694725.1	K00860	adenylylsulfate kinase [EC:2.7.1.25]	***H_2S production***
cysN	MBV6694726.1	K00956	sulfate adenylyltransferase subunit 1 [EC:2.7.7.4]	
cysD	MBV6694727.1	K00957	sulfate adenylyltransferase subunit 2 [EC:2.7.7.4]	
cysG	MBV6694728.1	K02302	sirohydrochlorin ferrochelatase [EC:2.1.1.107 1.3.1.76 4.99.1.4]	
cysH	MBV6694730.1	K00390	phosphoadenosine phosphosulfate reductase [EC:1.8.4.8 1.8.4.10]	
cysI	MBV6694731.1	K00381	sulfite reductase (NADPH) hemoprotein beta-component [EC:1.8.1.2]	
cysJ	MBV6694732.1	K00380	sulfite reductase (NADPH) flavoprotein alpha-component [EC:1.8.1.2]	
cth	MBV6691019.1	K01758	cystathionine gamma-lyase [EC:4.4.1.1]	
cbs	MBV6691020.1	K01697	cystathionine beta-synthase [EC:4.2.1.22]	
ilvM	MBV6694051.1	K11258	acetolactate synthase II small subunit [EC:2.2.1.6]	***Acetoin and 2,3-butanediol production***
ilvH	MBV6694244.1	K01653	acetolactate synthase I/III small subunit [EC:2.2.1.6]	
ilvB	MBV6694245.1	K01652	acetolactate synthase I/II/III large subunit [EC:2.2.1.6]	
budA	MBV6692473.1	K01575	acetolactate decarboxylase [EC:4.1.1.5]	
butA	MBV6694547.1	K18009	diacetyl reductase	
ubiC	MBV6692171.1	K03181	chorismate lyase [EC:4.1.3.40]	
metH	MBV6692197.1	K00548	5-methyltetrahydrofolate--homocysteine methyltransferase [EC:2.1.1.13]	***4-hydroxybenzoate, methanethiol, isoprene, sodorifn production***
gcpE	MBV6693643.1	K03526	(E)-4-hydroxy-3-methylbut-2-enyl-diphosphate synthase [EC:1.17.7.1 1.17.7.3]	
ispE	MBV6691414.1	K00919	4-diphosphocytidyl-2-C-methyl-D-erythritol kinase [EC:2.7.1.148]	
sodA	MBV6694136.1	K01823	isopentenyl-diphosphate Delta-isomerase [EC:5.3.3.2]	
sodB	MBV6695426.1	K01662	1-deoxy-D-xylulose-5-phosphate synthase [EC:2.2.1.7]	
gacS	MBV6694203.1	K07678	sensor histidine kinase ***GacS***	***Biosynthesis and transportation of diverse antibiotics***
gacA	MBV6691275.1	K07689	response regulator GacA	
hcnC	MBV6690484.1	-	hydrogen cyanide synthase	***HCN production***
chiA	MBV6693592.1	K01183	chitinase [EC:3.2.1.14]	
gbpA	MBV6692519.1	-	chitin-binding protein	
chiB	MBV6694019.1	-	chitinase [EC:3.2.1.14]	
chbG	MBV6694707.1	-	chitobiose	***Lytic and antioxidant enzymes***
amyA	MBV6690782.1	K01176	alpha-amylase [EC:3.2.1.1]	
apeE	MBV6693525.1	K12686	outer membrane lipase/esterase	
yhbV	MBV6693058.1	-	protease	
yegQ	MBV6693691.1	K08303	protease	
katG	MBV6692224.1	K03782	catalase-peroxidase [EC:1.11.1.21]	
katE	MBV6692316.1	K03781	catalase [EC:1.11.1.6]	
sodA	MBV6693541.1	K04564	superoxide dismutase [Mn]	
sodB	MBV6694141.1	K04564	superoxide dismutase [Fe]	
sodC	MBV6694127.1	K04565	superoxide dismutase, Cu-Zn family [EC:1.15.1.1]	
gstA	MBV6692140	K00799	glutathione S-transferase [EC:2.5.1.18]	
gpo	MBV6694168.1	K00432	glutathione peroxidase [EC:1.11.1.9]	
gsiA	MBV6690934.1	K13892	glutathione transport system ATP-binding protein	
gsiB	MBV6690935.1	K13889	glutathione transport system substrate-binding protein	
gsiC	MBV6690936.1	K13890	glutathione transport system permease protein	
gsiD	MBV6690937.1	K13891	glutathione transport system permease protein	
gor	MBV6693993.1	K00383	glutathione reductase (NADPH) [EC:1.8.1.7]	
oxyR	MBV6695241.1	K04761	LysR family transcriptional regulator, hydrogen peroxide-inducible genes activator	
rpoS	MBV6695493.1	K03087	RNA polymerase nonessential primary-like sigma factor	
uspA	MBV6693970.1	K06149	universal stress protein A	
uspB	MBV6693969.1	K06144	universal stress protein B	

uspC	MBV6692955.1	K14064	universal stress protein C	
uspE	MBV6691522.1	K14055	universal stress protein E	
uspG	MBV6691261.1	K11932	universal stress protein G	
flgN	MBV6692976.1	K02399	flagellar biosynthesis protein FlgN	***Flagellar assembly***
flgM	MBV6692977.1	K02398	negative regulator of flagellin synthesis FlgM	
flgA	MBV6692978.1	K02386	flagellar basal body P-ring formation protein FlgA	
flgB	MBV6692979.1	K02387	flagellar basal-body rod protein FlgB	
flgC	MBV6692980.1	K02388	flagellar basal-body rod protein FlgC	
flgD	MBV6692981.1	K02389	flagellar basal-body rod modification protein FlgD	
flgE	MBV6692982.1	K02390	flagellar hook protein FlgE	
flgF	MBV6692983.1	K02391	flagellar basal-body rod protein FlgF	
flgG	MBV6692984.1	K02392	flagellar basal-body rod protein FlgG	
flgH	MBV6692985.1	K02393	flagellar L-ring protein FlgH	
flgI	MBV6692986.1	K02394	flagellar P-ring protein FlgI	
flgJ	MBV6692987.1	K02395	peptidoglycan hydrolase FlgJ	
flgK	MBV6692988.1	K02396	flagellar hook-associated protein 1	
flgL	MBV6692989.1	K02397	flagellar hook-associated protein 3 FlgL	
fliR	MBV6692991.1	K02421	flagellar biosynthesis protein FliR	
fliQ	MBV6692992.1	K02420	flagellar biosynthesis protein FliQ	
fliP	MBV6692993.1	K02419	flagellar biosynthesis protein FliP	
fliO/fliZ	MBV6692994.1	K02418	flagellar protein FliO/FliZ	
fliN	MBV6692995.1	K02417	flagellar motor switch protein FliN	
fliM	MBV6692996.1	K02416	flagellar motor switch protein FliM	
fliL	MBV6692997.1	K02415	flagellar protein FliL	
fliK	MBV6692998.1	K02414	flagellar hook-length control protein FliK	
fliJ	MBV6692999.1	K02413	flagellar protein FliJ	
fliI	MBV6693000.1	K02412	flagellum-specific ATP synthase [EC:7.4.2.8]	
fliH	MBV6693001.1	K02411	flagellar assembly protein FliH	
fliG	MBV6693002.1	K02410	flagellar motor switch protein FliG	
fliF	MBV6693003.1	K02409	flagellar M-ring protein FliF	
fliE	MBV6693004.1	K02408	flagellar hook-basal body complex protein FliE	
fliT	MBV6693006.1	K02423	flagellar protein FliT	
fliS	MBV6693007.1	K02422	flagellar secretion chaperone FliS	
fliD	MBV6693008.1	K02407	flagellar hook-associated protein 2	
fliC	MBV6693009.1	K02406	flagellin	
fliA	MBV6693010.1	K02405	RNA polymerase sigma factor FliA	
fliZ	MBV6693011.1	K02425	regulator of sigma S factor FliZ	
fliY	MBV6693013.1	K02424	L-cystine transport system substrate-binding protein	
flhD	MBV6692957.1	K02403	flagellar transcriptional activator FlhD	
flhC	MBV6692958.1	K02402	flagellar transcriptional activator FlhC	
flhB	MBV6692971.1	K02401	flagellar biosynthesis protein FlhB	
flhA	MBV6692972.1	K02400	flagellar biosynthesis protein FlhA	
flhE	MBV6692973.1	K03516	flagellar protein FlhE	
motA	MBV6692959.1	K02556	chemotaxis protein MotA	***Chemotaxis***
motB	MBV6692960.1	K02557	chemotaxis protein MotB	
cheA	MBV6692961.1	K03407	two-component system, chemotaxis family, sensor kinase CheA [EC:2.7.13.3]	
cheW	MBV6692962.1	K03408	purine-binding chemotaxis protein CheW	
cheR	MBV6692965.1	K00575	chemotaxis protein methyltransferase CheR [EC:2.1.1.80]	
cheB	MBV6692966.1	K03412	two-component system, chemotaxis family, protein-glutamate methylesterase/glutaminase [EC:3.1.1.61 3.5.1.44]	
cheY	MBV6692967.1	K03413	two-component system, chemotaxis family, chemotaxis protein CheY	
cheZ	MBV6692968.1	K03414	chemotaxis protein CheZ	
tsr	MBV6692963.1	K05874	methyl-accepting chemotaxis protein I, serine sensor receptor	
tap	MBV6692964.1	K05877	methyl-accepting chemotaxis protein IV, peptide sensor receptor	
mcp	MBV6690869.1	K03406	methyl-accepting chemotaxis protein	
pilT	MBV6694347.1	K02669	twitching motility protein PilT	***Adhesive structure***

hofB	MBV6694216.1	K02504	protein transport protein HofB	
hofC	MBV6694217.1	K02505	protein transport protein HofC	
hofM	MBV6694875.1	K12288	pilus assembly protein HofM	
hofO	MBV6694877.1	K12290	pilus assembly protein HofO	
hofP	MBV6694878.1	K12291	pilus assembly protein HofP	
hofQ	MBV6694879.1	K02507	protein transport protein HofQ	
ppdC	MBV6693326.1	K02681	prepilin peptidase dependent protein C	
ppdB	MBV6693328.1	K02680	prepilin peptidase dependent protein B	
ppdA	MBV6693329.1	K02679	prepilin peptidase dependent protein A	
bcsA	MBV6693603.1	K00694	cellulose synthase (UDP-forming) [EC:2.4.1.12]	***Exopolysaccharide's biosynthesis***
bcsB	MBV6693604.1	K20541	cellulose synthase operon protein B	
bcsZ	MBV6693605.1	K20542	endoglucanase [EC:3.2.1.4]	
bcsC	MBV6693606.1	K20543	cellulose synthase operon protein C	
bcsE	MBV6693600.1	-	cellulose biosynthesis protein BcsE	
bcsF	MBV6693599.1	-	cellulose biosynthesis protein BcsF	
bcsG	MBV6693598.1	-	cellulose biosynthesis protein BcsG	
wza, gfcE	MBV6690971.1	K01991	polysaccharide biosynthesis/export protein	
etk-wzc	MBV6693117.1	K16692	tyrosine-protein kinase Etk/Wzc [EC:2.7.10.-]	
wzb	MBV6690972.1	K01104	phosphotyrosine protein phosphatase family	
csgG	MBV6690785.1	-	Curli production assembly/transport component CsgG	
tqsA	MBV6691312.1	K11744	AI-2 transport protein TqsA	***Quorum sensing***
luxS	MBV6694697.1	K07173	S-ribosylhomocysteine lyase [EC:4.4.1.21]	
tolC	MBV6691974.1	K12340	outer membrane protein	***Secretion system***
gspS	MBV6691946.1	K02465	general secretion pathway protein S	***Type II***
gspL	MBV6691949.1	K02461	general secretion pathway protein L	
gspK	MBV6691950.1	K02460	general secretion pathway protein K	
gspJ	MBV6691951.1	K02459	general secretion pathway protein J	
gspI	MBV6691952.1	K02458	general secretion pathway protein I	
gspG	MBV6691954.1	K02456	general secretion pathway protein G	
gspF	MBV6691955.1	K02455	general secretion pathway protein F	
gspE	MBV6691956.1	K02454	general secretion pathway protein E [EC:7.4.2.8]	
gspD	MBV6691957.1	K02453	general secretion pathway protein D	
secE	MBV6695377.1	K03073	preprotein translocase subunit SecE	***Sec-SRP***
secG	MBV6693040.1	K03075	preprotein translocase subunit SecG	
secA	MBV6694223.1	K03070	preprotein translocase subunit SecA [EC:7.4.2.8]	
secM	MBV6694224.1	K13301	secretion monitor	
secY	MBV6694952.1	K03076	preprotein translocase subunit SecY	
secB	MBV6694821.1	K03071	preprotein translocase subunit SecB	
secD	MBV6695411.1	K03072	preprotein translocase subunit SecD	
secF	MBV6695412.1	K03074	preprotein translocase subunit SecF	
yajC	MBV6695410.1	K03210	preprotein translocase subunit YajC	
yidC	MBV6693489.1	K03217	YidC/Oxa1 family membrane protein insertas	
ftsY	MBV6694597.1	K03110	fused signal recognition particle receptor	
ffh	MBV6694694.1	K03106	signal recognition particle subunit SRP54 [EC:3.6.5.4]	
vgrG	MBV6691215.1	K11904	type VI secretion system secreted protein VgrG	***Type VI***
impL	MBV6691224.1	K11891	type VI secretion system protein ImpL	
vasJ	MBV6691225.1	K11910	type VI secretion system protein VasJ	
impG	MBV6691226.1	K11896	type VI secretion system protein ImpG	
impH	MBV6691227.1	K11895	type VI secretion system protein ImpH	
vasD	MBV6691228.1	K11906	type VI secretion system protein VasD	
vasL	MBV6691231.1	K11911	type VI secretion system protein VasL	
impK	MBV6692947.1	K11892	type VI secretion system protein ImpK	
impJ	MBV6692948.1	K11893	type VI secretion system protein ImpJ	
vasD	MBV6692949.1	K11906	type VI secretion system protein VasD	
yidE	MBV6691032.1	K07085	putative transport protein	

hspA	MBV6694303.1	K04043	HSPA9 molecular chaperone
yidR	MBV6693508.1	-	biopolymer transporter Tol
yidQ	MBV6693509.1	-	protein of unknown function (DUF1375)

Table S3. Genes related to CAZymes in the *S. quinivorans* KP32 strain.

**Family of enzymes**	**CBM**	**GH**	**GT**	**AA**	**CE**
Number of gene	21	79	57	6	5

CBM. Carbohydrate-Binding Module; GH. Glycoside Hydrolase; GT. Glycosyl Transferase; AA. Auxiliary Activity; CE. Carbohydrate Esterase.

Table S4. CAZymes involved in plant and fungal cell wall degradation identified in the *S. quinivorans* KP32 genome.

**Substrate**	**Family enzymes**	**Annotation**	**Copy number**
Hemicellulose	GH36	α-galactosidase (EC 3.2.1.22)	2
Pectin	GH28	polygalacturonase (EC 3.2.1.15)	1
Polysaccharides	GH13	α-amylase (EC 3.2.1.1)	10
Peptidoglycans	GH23	lysozyme type G (EC 3.2.1.17)	10
Polysaccharides	GH65	α,α-trehalase (EC 3.2.1.28)	2
Chitooligosaccharide	CE4	chitooligosaccharide deacetylase (EC 3.5.1.-)	1
Polysaccharides	CE9	N-acetylglucosamine 6-phosphate deacetylase (EC 3.5.1.25)	2

Table S5. Ability of endophytic strain *S. quinivorans* KP32 to use organic compounds as sole sources of carbon and energy

**Organic compounds**	**Strain KP32**
Glucose	+++++
Arabinose	++
Rhamnose	-
Mannose	++++
Trehalose	+++
Succinic acid	+++
4-Hydroxyphenylacetic acid	++
Fumaric acid	++
Benzoic acid	+++
Mannitol	++
Citric acid	++
p-Coumaric acid	+

(+) capability, (−) lack of capability
